# The IgCAM BT-IgSF (IgSF11) Is Essential for Connexin43-Mediated Astrocyte–Astrocyte Coupling in Mice

**DOI:** 10.1523/ENEURO.0283-23.2024

**Published:** 2024-03-06

**Authors:** Laura Pelz, Laura Dossou, Nine Kompier, René Jüttner, Gabrielle Siemonsmeier, Niklas Meyer, Elijah D. Lowenstein, Ines Lahmann, Helmut Kettenmann, Carmen Birchmeier, Fritz G. Rathjen

**Affiliations:** ^1^Max-Delbrück-Center for Molecular Medicine, Berlin DE-13092, Germany; ^2^Shenzhen Institute of Advanced Technology, Chinese Academy of Sciences, Shenzhen 518055, China; ^3^NeuroCure Cluster of Excellence, Charité Universitätsmedizin Berlin, Corporate Member of Freie Universität Berlin and Humboldt-Universität zu Berlin, Berlin 10117, Germany

**Keywords:** astrocyte coupling, astrocyte, cell adhesion, connexin43, gap junction, IgCAMs

## Abstract

The type I transmembrane protein BT-IgSF is predominantly localized in the brain and testes. It belongs to the coxsackievirus and adenovirus receptor subgroup of Ig cell adhesion proteins, which are hypothesized to regulate connexin expression or localization. Here, we studied the putative link between BT-IgSF and connexins in astrocytes, ependymal cells, and neurons of the mouse. Global knock-out of BT-IgSF caused an increase in the clustering of connexin43 (Gja1), but not of connexin30 (Gjb6), on astrocytes and ependymal cells. Additionally, knock-out animals displayed reduced expression levels of connexin43 protein in the cortex and hippocampus. Importantly, analysis of biocytin spread in hippocampal or cortical slices from mature mice of either sex revealed a decrease in astrocytic cell–cell coupling in the absence of BT-IgSF. Blocking either protein biosynthesis or proteolysis showed that the lysosomal pathway increased connexin43 degradation in astrocytes. Localization of connexin43 in subcellular compartments was not impaired in astrocytes of BT-IgSF mutants. In contrast to connexin43, the localization and expression of connexin36 (Gjd2) on neurons were not affected by the absence of BT-IgSF. Overall, our data indicate that the IgCAM BT-IgSF is essential for correct gap junction–mediated astrocyte–astrocyte cell communication.

## Significance Statement

Astrocytes regulate a variety of physiological processes in the developing and adult brain that are essential for proper brain function. Astrocytes form extensive networks in the brain and communicate via gap junctions. Disruptions of gap junction coupling are found in several diseases such as neurodegeneration or epilepsy. Here, we demonstrate that the cell adhesion protein BT-IgSF is essential for gap junction–mediated coupling between astrocytes in the cortex and hippocampus.

## Introduction

BT-IgSF (brain- and testis-specific Ig superfamily protein, also known as IgSF11) is a cell adhesion protein belonging to a small subgroup of IgCAMs consisting of coxsackievirus and adenovirus receptor (CAR), endothelial cell-selective adhesion molecule, and CAR-like membrane protein (CLMP). The group shares a similar overall domain organization with an N-terminal V-type and a C2-type Ig domain and a highly related amino acid sequence (Raschperger et al., 2004; Rathjen, 2020; Xie et al., 2021; Owczarek et al., 2023). Initially, BT-IgSF was described as a novel IgSF member that was preferentially expressed in the brain and testis (Suzu et al., 2002). Independently of this report, BT-IgSF was also found to be upregulated in intestinal-type gastric cancers and termed IgSF11 (Katoh and Katoh, 2003). Moreover, it was also termed V-set and immunoglobulin domain containing 3, abbreviated VSIG-3, due to its binding to VISTA (Wang et al., 2019). The BT-IgSF gene is located on chromosome 3 in humans and chromosome 16 in mice. The cytoplasmic segment of BT-IgSF contains a PDZ-binding motif at its C-terminus that interacts with the scaffolding protein PSD95 (Jang et al., 2015). Adhesion assays with heterologous cells showed that BT-IgSF promotes homotypic cell binding (Harada et al., 2005; Eom et al., 2012).

So far, the function of BT-IgSF (IgSF11) has been studied in neurons, Sertoli cells, and germ cells of the testes, during osteoclast differentiation and in the organization of pigment cells in fish (Eom et al., 2012; Jang et al., 2015; Singh and Nusslein-Volhard, 2015; Ahi and Sefc, 2017; Pelz et al., 2017; Kim et al., 2020; Chen et al., 2021; Hayano et al., 2021; G. M. Kim et al., 2023; H. Kim et al., 2023). Knockdown studies using cultured mouse hippocampal neurons indicated that BT-IgSF is implicated in synaptic transmission through a tripartite interaction with PSD95 and AMPA receptors (Jang et al., 2015). Consequently, global BT-IgSF–deficient mice displayed a moderately decreased excitatory synaptic strength in the dentate gyrus (DG) and long-term potentiation in hippocampal CA1 neurons and behavioral deficits (Jang et al., 2015; Montag et al., 2023). In another study analyzing neurons, BT-IgSF (IgSF11) was found to regulate chandelier cell axon innervation of pyramidal neuron initial axon segments (Hayano et al., 2021).

In the murine testis, BT-IgSF is expressed in the Sertoli cells at the blood–testis barrier, a structure that opens and closes to allow the passage of germ cells. In a global knock-out, the absence of BT-IgSF causes a malfunction of the blood–testis barrier leading to male infertility due to the mislocalization of connexin43. Connexin43 was found throughout the seminiferous epithelium instead of being restricted to the blood–testis barrier as it is in wild-type animals. Therefore, BT-IgSF might regulate the localization or activity of connexin43 in the Sertoli cells (Pelz et al., 2017). In line with this finding, connexin43 was found to play an essential role in tight junction reassembly at the blood–testis barrier during its restructuring processes (Li et al., 2010). A critical role of BT-IgSF in regulating the organization of pigment cells into stripes along the dorsoventral or anteroposterior body axes was observed in zebrafish and *Neolamprologus meeli* (Eom et al., 2012; Singh and Nusslein-Volhard, 2015; Ahi and Sefc, 2017). Similar irregular patterns of chromatophores were described in zebrafish with mutations in connexin41.8 and connexin39.4, suggesting a functional link between BT-IgSF and connexins. This link might also be anticipated from the mislocalization of connexin43 in the Sertoli cells of BT-IgSF knock-out mice (Haffter et al., 1996; Watanabe et al., 2006; Watanabe and Kondo, 2012; Irion et al., 2014; Pelz et al., 2017). Together, these data suggest an essential function of BT-IgSF in regulating the localization or activity of connexins as shown for other members of the CAR subgroup.

To further investigate the functional interaction between BT-IgSF and connexin43, we investigated its role in the brain on glial cells. Here, we show that BT-IgSF is strongly localized on the surface of astrocytes and ependymal cells in addition to its previously described neuronal expression (Suzu et al., 2002; Jang et al., 2015; Higashine et al., 2018). In the absence of BT-IgSF in a global mouse knock-out, we observed that the localization of connexin43 on astrocytes and ependymal cells was aberrantly clustered. Additionally, we uncovered a severe reduction in connexin43 on astrocytes at the protein level. Consequently, dye-loading experiments revealed reduced diffusion within the astrocytic network in the hippocampus as well as in the cortex of mutant mice. We discuss these findings in the context of the function of the related proteins CLMP and CAR, which also affect the expression and localization of connexin43 and connexin45 in smooth muscle cells of the intestine and cardiomyocytes, respectively (Lim et al., 2008; Lisewski et al., 2008; Langhorst et al., 2018; Rathjen, 2020; Matthaeus et al., 2022).

## Material and Methods

### Mice

The global knock-out of BT-IgSF (B6-Igsf11^tm1e(KOMP)Wtsi^/FGR) and its genotyping have been described elsewhere (Pelz et al., 2017). Breeding was either from tg/wt to tg/wt or tg/tg (female) to wt/tg (male). Heterozygous BT-IgSF mice did not differ from wt/wt mice (Pelz et al., 2017). Cx36-deficient mice (B6.129P2-Gjd2tm1Kwi/Cnrm; EM:00326) were obtained from the European Mouse Mutant Archive and genotyped as described (Güldenagel et al., 2001). Animals were housed on a 12/12 h light/dark cycle with free access to food and water. The animal procedures were performed according to the guidelines from directive 2010/63/EU of the European Parliament on the protection of animals used for scientific purposes. All experiments were approved by the local authorities (LaGeSO; numbers T0313/97, X9007/16, X9008/20, O 0038/08, and H0027/20). Wild-type littermates served as controls.

### Cell culture, antibodies, and immunocytochemistry

Hippocampal cells were prepared from postnatal day 2 or 3 mice and cultured on poly-d-lysine–coated coverslips in 24-well plates at a density of 7.5 × 10^4^ cells/ml in Neurobasal (Invitrogen) supplemented with B27 (Invitrogen) and 10% FCS (Invitrogen) for 10 d. Every third day half of the culture medium was removed and replaced by Neurobasal/B27 without FCS. Glial cells from postnatal day 2/3 cortices or hippocampi were prepared according to standard procedures and were maintained in DMEM supplemented with 10% FCS and penicillin/streptomycin in 75 cm^2^ flasks until confluent (McCarthy and de Vellis, 1980). Then, astrocytes were detached by trypsin/EDTA treatment and further grown in multiple 24-well clusters (200,000 cells per well) for cycloheximide or chloroquine (CQ) treatment (see below) or on poly-d-lysine coated coverslips (20,000 cells per 12 mm diameter coverslip) for staining with antibodies to specific intracellular compartments (see below). Cells of each 24-well cluster were extracted as described below and equal amounts were analyzed by Western blotting (see below). For immunocytochemistry, cells were fixed in 4% paraformaldehyde/PBS for 10 min and washed with PBS/1% BSA. For staining of cryosections (thickness 12–16 µm) by indirect immunofluorescence, adult mice were transcardially perfused with 50 ml PBS followed by 50 ml 4% paraformaldehyde. Brains were postfixed for 5 h and then transferred into 15% followed by 30% (w/v) sucrose in PBS to obtain cryoprotection. Primary and fluorophore-conjugated secondary antibodies were applied in a blocking solution (5% goat serum, 1% BSA, 0.1% Triton X-100 in PBS). Mouse monoclonal antibodies were incubated on sections using the MOM immunodetection kit from Vector Laboratories (BMK-2202). Sections or monolayer cultures were counterstained with the nuclear stain DAPI at 1 µg/ml. For antibodies, see Table 1.

**Table 1.** Antibodies for immunohistochemistry, immunocytochemistry, and Western blotting

Antibody	IHC and ICC	Western blots	Source
mAb γ-adaptin RRID: AB_397768	1 µg/ml		BD Transduction Laboratories, #610385
Rabbit anti–mBT-IgSF-Fc (Rb95 or Rb96) IgG	1–3 µg/ml	1 µg/ml	Pelz et al. (2017)
Rabbit anti-mCAR	1 µg/ml		Patzke et al. (2010)
mAb anti-connexin43 RRID: AB_397473	1 µg/ml		BD Transduction Laboratories, #610061
Rabbit anti-connexin43 RRID: AB_2294590	1:200	1:1,000	Cell Signaling Technology, #3512
Rabbit anti-connexin30 RRID: AB_2533979	1 µg/ml	0.5 µg/ml	Invitrogen, #71-2200
Rabbit anti-connexin36 RRID: AB_2533260		1 µg/ml	Zymed/Invitrogen, #36-4600
mAb connexin36 (H9)		1 µg/ml	Santa Cruz sc-398063
Rabbit anti–m-connexin36 (RbB5)	0.5 µg/ml	0.5 µg/ml	This study
mAb 2H3 anti-neurofilament RRID: AB_531793	7.5 µg/ml		mAb 2H3 Developmental Studies Hybridoma Bank
mAb 8F6.2 connexin35/36 RRID: AB_94632		1 µg/ml	Millipore, MAB3045
mAb ZO-1 RRID: AB_2533147	1:200		Invitrogen, #339100
Guinea pig anti-GFAP RRID: AB_10641162	1:500	1:1,000	Synaptic Systems, #173004
Goat anti-GFAP RID: AB_641021	1:200	1:1,000	Santa Cruz Biotechnology, #sc-6170
Rabbit anti-GFAP RRID: AB_10013382	1:1,000		Dako, Z0334
mAb GM130 RRID: AB_398142	1 µg/ml		BD Transduction Laboratories, #610823
mAb LAMP-1 (1D4B) RRID: AB_2134495	1 µg/ml		Santa Cruz Biotechnology, sc-19992
mAb anti-MAP2a/b (clone AP20)	1:500	1:1,000	Dianova, #DLN-08578
Rabbit anti–ZO-1 RRID: AB_2533456	1:100		Invitrogen, #40-2200
mAb anti-GAPDH (1D4) RRID: AB_10077627		0.5 µg/ml	Novus Biologicals, #NB300-221
mAb anti-clathrin (heavy chain) RRID: AB_397865		1:1,000	BD Transduction Laboratories, #610499
Goat anti-rabbit Cy3	1:400–1:1,000		Dianova
Goat anti-mouse Alexa488	1:400–1:1,000		Molecular Probes
Rabbit anti-goat Alexa488	1:500–1:1,000		Dianova
Goat anti-rabbit HRP		1:20,000	Dianova
Goat anti-mouse HRP		1:20,000	Dianova

IHC, immunohistochemistry; ICC, immunocytochemistry.

Microscopic images were obtained at room temperature by confocal imaging using a Carl Zeiss LSM 700 Laser Scanning Microscope equipped with ZEN 2011 software and the following lenses: a Plan-Neofluar 20×/0.30 NA objective, a Plan-Achromat 40×/1.3 Oil, a Plan-Achromat 63×/1.40 NA oil objective, or a Plan-Apochrome 100×/1.40 oil objective (all from Carl Zeiss MicroImaging). The figures were assembled using Illustrator CS5 (Adobe).

To quantify connexin43 clusters, confocal images were taken with a 63× objective from cryosections and a 100× objective for cultured astrocytes. Quantification of connexin43 clusters was done by Fiji software setting the threshold to RenyiEntropy routine; clusters were accepted between 0.05 and 1.00 µm^2^ (2–40 pixels). Primary branches of astrocytes and the density of astrocytes were counted from hippocampal slices stained by anti-GFAP- and DAPI-positive cells.

Analysis of localizations or colocalizations of connexin43 to the intracellular compartments was done with antibodies to γ-adaptin, LAMP-1, ZO-1, GM130, to phalloidin-Alexa594 (6.6 µM; 1:500; Thermo Fisher Scientific #A12381), or to BT-IgSF (Rb96; Table 1) on 4% paraformaldehyde/PBS-fixed (5 min on ice) on wild-type and knock-out astrocyte cultures. Assessment of cell surface localization of connexin43 cluster on knock-out astrocytes was done by life staining of astrocytes with WGA-488 (1 µg/ml; Thermo Fisher Scientific W11261) at 4°C for 45 min followed by washing, PFA fixation, and solubilization with 0.1% Triton X-100. Then, rabbit antibodies to connexin43 were added as described above. Images were obtained by confocal microscopy using the 100× oil objective mentioned above and were analyzed using Fiji/ImageJ. Calculation of the Pearson correlation was determined by Coloc 2–derived intensity-based correlation analysis from regions of interest around connexin43 clusters (10 µm circles). Costes threshold regression was applied and Pearson's *R*-value (P) above the threshold was used, and data were accepted with a Costes *p*-value of >0.95. Mann–Whitney *U* test (GraphPad Prism 6.07) was used to compare wild-type with knock-out values.

### Generation of a connexin36 fusion protein and polyclonal antibodies to connexin36

Since several commercial anti-connexin36 antibodies were of limited use in our hands, we generated polyclonal anti-mouse connexin36 antibodies in rabbits using a fusion protein that comprised the second cytoplasmic segment of mouse connexin36 (amino acid residues 99–197) attached to a histidine stretch. The cDNA of this segment was synthesized by Invitrogen, cloned into plasmid pMA-RQ, and further subcloned into the bacterial expression vector pET-14b (Novagen/EMD Millipore). The protein was expressed in Bl21 bacteria with the addition of IPTG to a final concentration of 1 mM. Bacteria were harvested by centrifugation and frozen at −80°C. Bacterial pellets were resuspended in ice-cold lysis buffer containing 2 M urea, 50 mM Tris, and 150 mM NaCl, pH 7.4, supplemented with protease blockers (aprotinin, PMSF, leupeptin, pepstatin). Unsolubilized material was removed by centrifugation, and the supernatant was precipitated by ammonium sulfate (50% saturation). The pellet was dialyzed against 10 mM Tris, pH 11, and run over an anion exchange column (DE52 Whatman). The unbound fraction was applied to an NTA column (Qiagen) and washed with PBS followed by 20 mM imidazole, and the bound protein was eluted by 200 mM imidazole. Purity was analyzed by 15% SDS-PAGE. Rabbits were injected with 100 µg protein in Freund's adjuvant at fortnightly intervals. The IgG fraction was obtained by protein A affinity chromatography (GE HealthCare) and further purified by affinity chromatography on an affinity column containing the abovementioned connexin36 segment protein coupled to CNBr-activated sepharose (14 mg to 2.5 g sepharose 4B; GE HealthCare). The specificity of the affinity-purified antibodies was tested on tissue extracts and cryostat sections (12 µm thick, mildly fixed with 1% paraformaldehyde/PBS for 1 min on ice) from wild-type or connexin36-deficient mice (Fig. 7).

### Biochemical methods

To obtain a crude membrane fraction from tissues of BT-IgSF knock-out or wild-type mice of different ages (as indicated in the figure legends), hippocampi or cortices were homogenized in 0.34 M sucrose supplemented with protease blockers [aprotinin (20 U/µl), leupeptin (5 mM), pepstatin (5 mM), PMSF (1 mM)]. Nuclei were pelleted at 200 × *g* for 10 min, and the resulting supernatant was centrifuged at 100,000 × *g* for 10 min to obtain a crude membrane pellet and cytoplasmic fraction in the supernatant. Membranes were stripped with 0.1 M diethylamine (pH11.5), supplemented with protease blockers to remove peripheral membrane proteins. The membrane fraction was first solubilized in 1% Triton X-100, and unsolubilized material was removed by centrifugation. The pellet was then solved in 1% SDS in PBS supplemented with protease blockers, and unsolubilized material was again removed by centrifugation (Musil and Goodenough, 1991). Protein concentrations were determined using the Bradford assay (Bio-Rad #500-0006) and spectrophotometric measurements. Equal amounts of proteins were loaded on SDS-PAGE for Western blotting, which was controlled by Ponceau protein stain and housekeeping proteins such as GAPDH, heavy chain of clathrin, or α-tubulin. Depending on the tissue or antibody, 10, 15, or 20 µg of protein was loaded per lane. Blots to identify BT-IgSF in cells or tissues SDS-PAGE was run without reducing agents. For the calculation of the molecular mass of connexin36 in neural tissues, the following molecular mass standards were used (in kDa): conalbumin, 76; BSA, 66; actin, 43; GAPDH, 36; and carbonic anhydrase, 31.

Cycloheximide chase experiments (100 µg/ml, Sigma, C-7698, dissolved in DMSO and diluted 1:500 in the incubation medium) were done with wild-type and BT-IgSF knock-out astrocytes cultures for times indicated in Figure 3, *G* and *H*. A total of 200,000 cells were grown in 24-well clusters in DMEM/10% FCS and washed two times with DMEM without FCS before the addition of cycloheximide. CQ experiments (100 µM; Sigma C6628, dissolved in PBS) to inhibit lysosomal degradation were done with wild-type and BT-IgSF knock-out astrocytes cultures for 4 h in DMEM without FCS (Wu et al., 2016; Schrezenmeier and Dörner, 2020). Treatment of wild-type astrocytes with tumor necrosis factor-α (1 ng/ml; Sigma T5944) or interferon-γ (10 ng/ml; Sigma I17001) were done in DMEM without FCS for 24 h. In these blocking experiments, cells were lysed in TBS (pH 7.4) with 1% SDS and 1 mM EDTA supplemented with protease blockers and boiled in SDS-PAGE sample buffer. Equal amounts of protein were loaded on 10% SDS-PAGE, blotted, and analyzed with rabbit anti-connexin43 or rabbit 96 to BT-IgSF. Equal loading was controlled by Ponceau protein stain and mAb to GAPDH. Quantification of connexin43 bands was done using Image Lab software (Bio-Rad). Total connexin43 expression was derived from the sum of connexin43 bands P0, P1, and P2 (bands P1 and P2 correspond to phosphorylated connexin43).

### RNAscope on hippocampus sections

In situ fluorescent hybridization was performed using the RNAscope multiplex fluorescent assay from ACDBio according to the manufacturer's instructions (Wang et al., 2012). Briefly, PFA-fixed hippocampus or midbrain sections of 20 µm thickness were obtained from P20 or P10 wild-type mice, respectively, and stored at −80°C until use. Sections were thawed at 37°C for 10 min and postfixed in 4% PFA in PBS for 15 min before washing in PBS and continuing with the manufacturer's instructions. Protease treatment was performed using protease IV. RNAscope probes against BT-IgSF (C1, 451131), GFAP (C2, 313211), vGlut1 (C2, 416631), and GAD65/67 (Gad1: C2, 400951; Gad2: C2, 439371) were used.

### Dye coupling in acute brain slices

To characterize the tracer spread within coupled astrocytic networks, acute brain slices containing the hippocampus or the cortex were prepared from 8- to 10-week-old knock-out or littermate controls of either sex as described previously (Maglione et al., 2010). In brief, mice were killed by cervical dislocation and decapitated, and their brains were carefully removed and mounted in a chamber with ice-cold bicarbonate-buffered artificial cerebrospinal fluid (ACSF), composed of the following (in mM): 134 NaCl, 2.5 KCl, 1.3 MgCl_2_, 2 CaCl_2_, 1.25 K_2_HPO_4_, 26 NaHCO_3_, and 10 d-glucose, pH 7.4. The buffer solution was continuously gassed with carbogen (95% O_2_, 5% CO_2_). Coronal slices of 250 µm were prepared at 4°C using a vibratome (HM 650V, Microm International) and stored in ACSF at room temperature (21–25°C) for up to 5 h.

Before dye filling, slices were incubated for 20 min in 1 µM sulforhodamine 101 (SR-101) at 35°C to label astrocytes. Astrocytes were identified by their SR-101 fluorescence at excitation and emission wavelengths of 555 and 585 ± 10 nm, respectively, using a 60× water immersion objective (Olympus). For recording and dye loading, a patch pipette (pulled from borosilicate glass, 1.5 mm outside diameter, 0.315 mm wall thickness) was filled with a solution containing 30 mM KCl, 1 mM MgCl_2_, 0.5 mM CaCl_2_, 100 mM potassium gluconate, 10 mM Hepes, 5 mM EGTA, 0.5% biocytin (Sigma-Aldrich), and 3 mM Na_2_ATP, pH 7.3. Lucifer yellow (10 µg/ml; Sigma-Aldrich) was added to the pipette solution, and intracellular access of the solution was confirmed by excitation at 495 nm and visualization at an emission wavelength of 510 ± 10 nm. The pipette resistance ranged from 5 to 8 MΩ. Cells were passively dialyzed via the patch pipette for 20 min. In order to confirm cell identity and vitality, membrane currents were recorded with a series of de- and hyperpolarizing voltage steps (10 mV each, filtered at 2.9 kHz) from a holding potential of −70 mV ranging from −160 to +50 mV for 50 ms), using an EPC 10 patch-clamp amplifier and TIDA 5.25 software (HEKA Elektronik), as described previously (Richter et al., 2014). Capacitive transients from the pipette were compensated online via the patch-clamp amplifier (C_fast_), whereas membrane capacity and series resistance (C_slow_) were not compensated. The calculated liquid junction potential of the used intracellular solutions was −8.858 mV using Patchers Power Tools (Mendez & Würriehausen) and Igor Pro 7 software (WaveMetrics). The calculated reversal potentials of astrocytes were corrected for the liquid junction potential. Only cells whose series resistance was not higher than 125% at the end of the dialysis phase compared with the beginning of the recording were taken into account for the following immunohistochemical experiments and the calculation of the membrane properties. After dye loading and patch-clamp recording, the pipette was carefully removed from the cell in order to disrupt the patch.

Slices were subsequently fixed in a solution of 4% paraformaldehyde in 0.1 M phosphate-buffered saline (pH 7.4) overnight at 4°C. After fixation, slices were incubated in a solution containing 2% Triton X-100, 2% BSA, and 5% normal donkey serum in Tris-buffered saline at pH 7.4 for 2 h at room temperature to permeabilize and to block nonspecific binding of the primary antibodies. Biocytin-filled networks were visualized with Cy3-conjugated streptavidin (1:200; Jackson ImmunoResearch). In addition, rabbit anti–BT-IgSF (Rb95) and guinea pig anti-GFAP antibodies were applied to label BT-IgSF and astrocytes, respectively. The floating slices were incubated with primary antibodies for 48 h at 4°C followed by secondary antibodies and DAPI. For additional antibodies, see Table 1.

Slices were rinsed and mounted with Aqua Poly/Mount (Polysciences). Images were acquired by a Leica DM TCS SPE confocal microscope (HC APO 20x/0.75; Leica) with Leica software (LCS Lite or LAS AF Lite, respectively). The step size between *z*-planes in the confocal stacks was about 1 µm, but the number of imaged planes in each stack varied from slice to slice. Images were analyzed by Fiji/ImageJ software, using the cell counter plugin and *z*-axis projection functions.

### Statistics

The significance of data was tested using nested *t* test, Mann–Whitney *U* test, unpaired *t* test with Welch’s correction, or one-way ANOVA using GraphPad Prism software (versions 6.07 and 10.1.2) after excluding the outliers using the outlier tests (ROUT, *Q* = 1%). Normality was tested using the D’Agostino–Pearson omnibus normality test or Shapiro–Wilk normality test.

## Results

### Absence of BT-IgSF disrupts the expression and localization of connexin43 in the hippocampus and on cultured astrocytes

**Figure 1.**
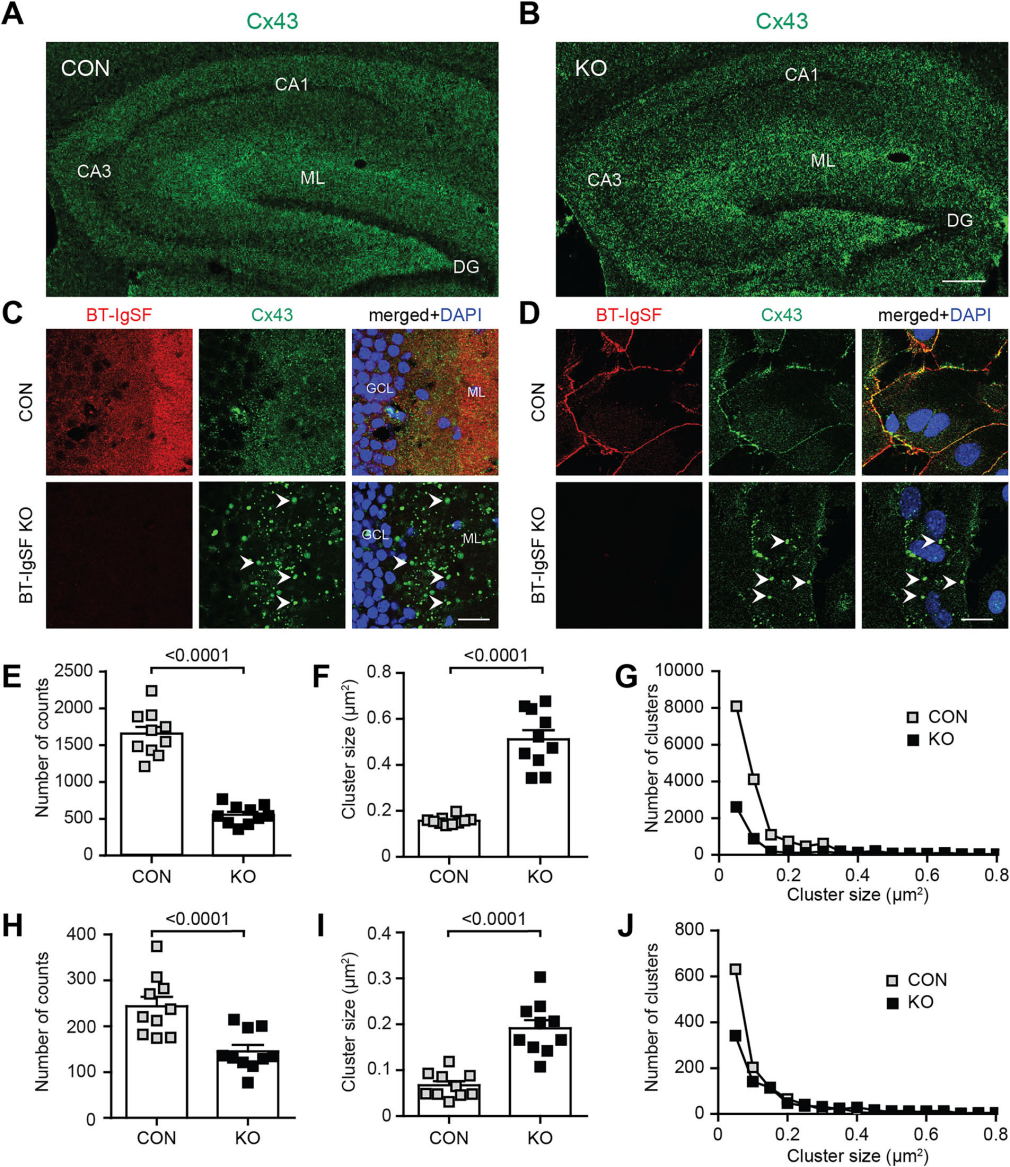
Impaired clustering of connexin43 in astrocytes in the absence of BT-IgSF. ***A***, ***B***, Overview of the location of connexin43 in coronal sections of the hippocampus from wild-type (***A***) and BT-IgSF–deficient (***B***) mice (10 weeks old). CA1, cornus ammonis 1; CA3, cornus ammonis 3; DG, dentate gyrus; GCL, granule cell layer; ML, molecular layer. Scale bar, 100 µm. ***C***, Higher magnification of the ML of the DG from adult wild-type and knock-out mice stained by mAb anti-connexin43, rabbit anti–BT-IgSF, and DAPI. Arrow heads indicate large connexin43 clusters. ***D***, Cortical astrocyte monolayer cultures from P3 wild-type or knock-out mice at DIV14 stained by mAb anti-connexin43, rabbit anti–BT-IgSF, and DAPI. Scale bar, 20 µm. ***E***, ***F***, Quantification of the connexin43 spots and cluster size on astrocytes in the hippocampus. In total, 1,652.5 ± 306.6 (mean ± SD) and 505.4 ± 128.1 (mean ± SD) connexin43 spots from wild-type and BT-IgSF−/−, respectively, from 10 cryosections (63× objective) of three specimens of each genotype were counted. A nested *t* test was applied. ***H***, ***I***, Quantification of the connexin43 spots and cluster size of connexin43 on cultured cortical astrocytes. Control, 243 ± 65.1 (mean ± SD); BT-IgSF−/−, 132.9 ± 43.9 spots from 10 view fields (100× objective) from three independent cultures per genotype were counted. Nested *t* test. ***G***, ***J***, Cluster size distribution of connexin43 versus cluster number in the hippocampus or on astrocytes in culture (***J***). Scale bar, 100 µm (***B***); 20 µm (***C*** and ***D***).

BT-IgSF was first described as an IgSF member that was preferentially expressed in the brain and testis (Suzu et al., 2002). BT-IgSF and related proteins of the CAR family of adhesion proteins have been proposed to modulate the localization of connexins (Rathjen, 2020). We tested whether the absence of BT-IgSF affects the localization of connexin43 expression on astrocytes as it does on the Sertoli cells in the testes (Pelz et al., 2017). In the mature brain, connexin43 is expressed by astrocytes and ependymal cells, but not by neurons. Connexin30 and connexin26 have also been detected in adult astrocytes, albeit at lower levels (Dermietzel et al., 1989; Nagy et al., 2004). Hippocampal sections as well as astrocyte monolayer cultures were labeled with rabbit anti-connexin43 antibodies. Microscopic images of hippocampal sections revealed an altered localization of connexin43 in BT-IgSF knock-out tissue (Fig. 1*A*,*B*). In the molecular layer (ML) of knock-out hippocampal tissue, a pronounced decrease in the number of connexin43 spots (31% of the wild-type), accompanied by a marked increase in the connexin43 spot size (average of 0.19–0.5 µm^2^ in wild-type and BT-IgSF−/− mice, respectively), was observed in high power magnifications (Fig. 1*C*,*E*,*F*). Similar observations on the clustering and number of connexin43 spots were made on cultured astrocytes, suggesting that BT-IgSF might control connexin43 localization even in the absence of neurons (Fig. 1*D*,*H*,*I*). Analysis of the distribution of the number of counts versus cluster size further highlights the difference between wild-type and BT-IgSF knock-outs (Fig. 1*G*,*J*). Taken together, our data indicate that the absence of BT-IgSF decreases the number of connexin43 spots but increases their clustering in astrocytes.

**Figure 2.**
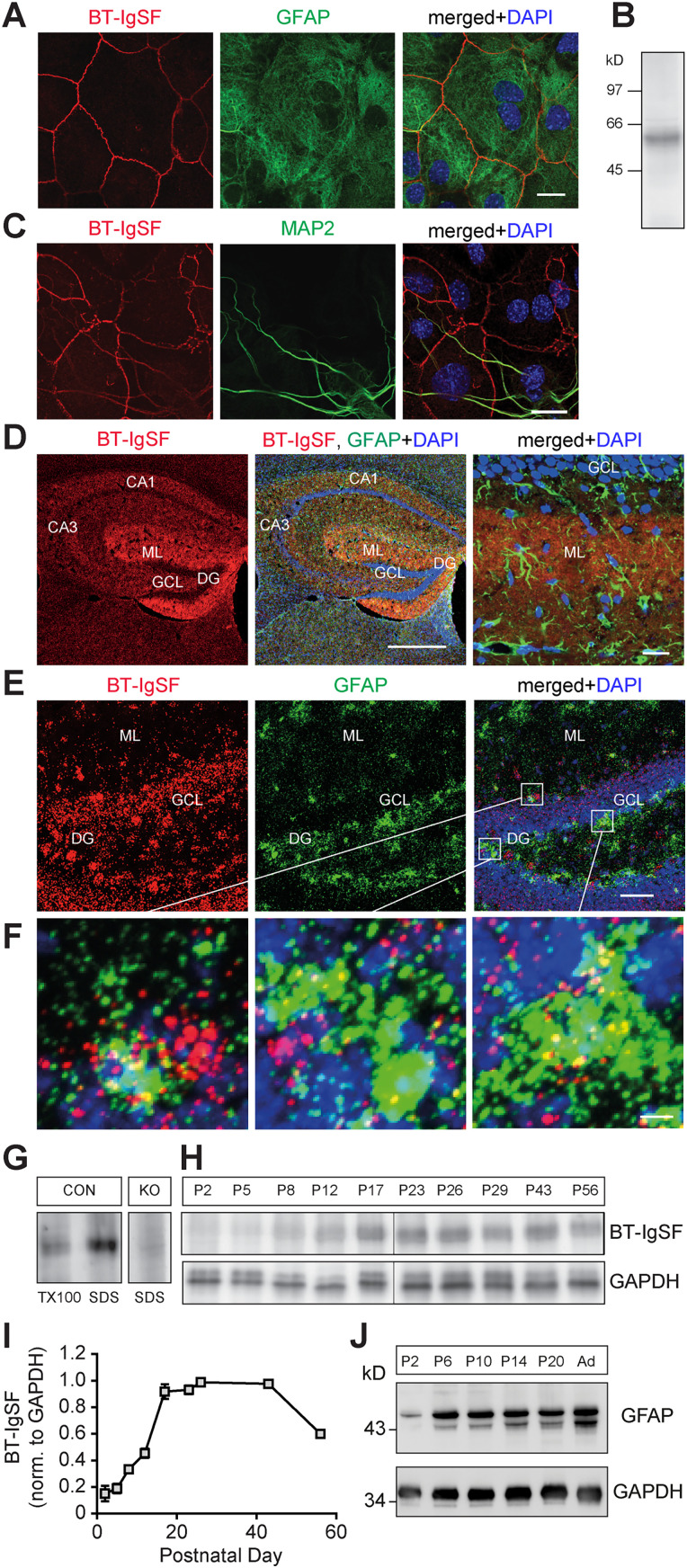
Expression of BT-IgSF on astrocytes (see Extended Data Fig. 2-1 for BT-IgSF antibody specificity and Extended Data Fig. 2-2 for BT-IgSF mRNA expression in neurons). ***A***, Localization of BT-IgSF in hippocampus cells from P2-old mice cultured for 10 d in vitro and stained by guinea pig antibodies to GFAP and rabbit anti–BT-IgSF. Three independent cultures were analyzed. Scale bar, 20 µm. ***B***, Western blot of extracts from an astrocyte culture using rabbit antibodies to BT-IgSF. Extracts from two cultures were mixed and three times analyzed by blots. Molecular mass markers are indicated at the left of the panel. ***C***, Cultured hippocampus cells were stained by antibodies to MAP2a/b and rabbit anti–BT-IgSF. Three cultures were analyzed. Scale bar, 30 µm. BT-IgSF is found on GFAP astrocytes but rarely detectable on MAP2a/b-positive neurons by antibodies. ***D***, Localization of BT-IgSF in a coronal hippocampus section from an adult mouse. BT-IgSF is primarily found in the ML and the subgranular zone. Higher magnification shows a widespread localization in the ML probably not restricted to a specific cell type. Five independent experiments were done. CA1, cornus ammonis 1; CA3, cornus ammonis 3; DG, dentate gyrus; GCL, granule cell layer; ML, molecular layer. Left and middle panels, scale bar, 1 mm. Right panel, scale bar, 20 µm. ***E***, RNAscope of a coronal section of the hippocampus at P20 showing expression of *Bt-igsf* mRNA in GFAP-positive astrocytes in the ML and hilus of the DG. Three independent sections were analyzed. Scale bar, 50 µm. ***F***, Higher magnifications of squares as indicated in ***E***. Scale bar, 5 µm. ***G***, Effective extraction of BT-IgSF from tissues requires SDS. Equal amounts of crude membrane fractions from the brain (P56) were extracted with 1% Triton X-100 or SDS. For the specificity of the antibody, knock-out brain tissue is shown. Three independent extractions were done. ***H***, ***I***, Different postnatal stages of brain extracts using SDS stained with anti–BT-IgSF are shown (*n* = 3). BT-IgSF is primarily found at advanced postnatal stages. Anti-GAPDH indicates loading. ***J***, For comparison, GFAP expression is shown.

We further investigated BT-IgSF expression on neural cells by immunocytochemistry of hippocampal cultures and brain sections. In monolayer cultures prepared from newborn hippocampal tissue, BT-IgSF was primarily localized on the surface of GFAP-positive astrocytes (Fig. 2*A*,*B* and Extended Data Fig. 2-1 on the specificity of the anti–BT-IgSF antibody) and was not or only weakly detected on MAP2-positive dendrites (Fig. 2*C*). However, single-molecule fluorescent in situ hybridization (RNAscope) experiments indicated that neurons express detectable amounts of *Bt-igsf* mRNA (Extended Data Fig. 2-2). Similarly, *Bt-igsf* mRNA was found in GFAP-positive cells in sections of the ML of the hippocampus (Fig. 2*E*,*F*), which is further supported by immunohistochemistry using polyclonal antibodies to the extracellular region of BT-IgSF (Fig. 2*D*). BT-IgSF protein expression increases as the brain matures postnatally and effective solubilization from crude membrane fractions requires ionic detergents such as SDS (Fig. 2*G*–*J*).

10.1523/ENEURO.0283-23.2024.f2-1 Figure 2-1 **Localization of BT-IgSF in the hippocampus at P14 and P80 and demonstration of the specificity of rabbit anti-BT-IgSF.** Coronal cryostat sections of the hippocampus at P14 (A) or P80 (B) were stained with anti-BT-IgSF (Rb95; 1 µg/ml). The dashed boxes indicate the position of the enlarged region shown below row 1 and 3. Absence of staining in BT-IgSF knockout tissues demonstrates specificity of rabbit antibodies to BT-IgSF. Two independent animals of each age were analyzed. CA1, cornus ammonis 1; CA3, Cornus ammonis 3; DG, dentate gyrus; GCL, granule cell layer; ML, molecular layer. Scale bar first and third row, 1 mm; scale bar second and fourth row, 20 µm. Download Figure 2-1, TIF file.

10.1523/ENEURO.0283-23.2024.f2-2 Figure 2-2 ***Bt-igsf* encoding mRNA is expressed in excitatory and inhibitory neurons of the hippocampus.** A) Overview of an RNA scope of *Bt-igsf* of a coronal section of a P20 hippocampus showing *Bt-igsf* mRNA encoding cells in all cell layers. Scale bar 500 µm. B) RNAscope of coronal sections of hippocampi at postnatal day 20 showing expression of *Bt-igsf* in GFAP-positive astrocytes (see also Figure 2F). C) RNAscope of coronal sections of hippocampi at postnatal day 20 showing expression of BT-IgSF in *vGlut1*-positive neurons. D) RNAscope of coronal sections of hippocampi at postnatal day 20 showing expression of *Bt-igsf* in *Gad65*-positive neurons. Three independent sections were analyzed. Download Figure 2-2, TIF file.

**Figure 3.**
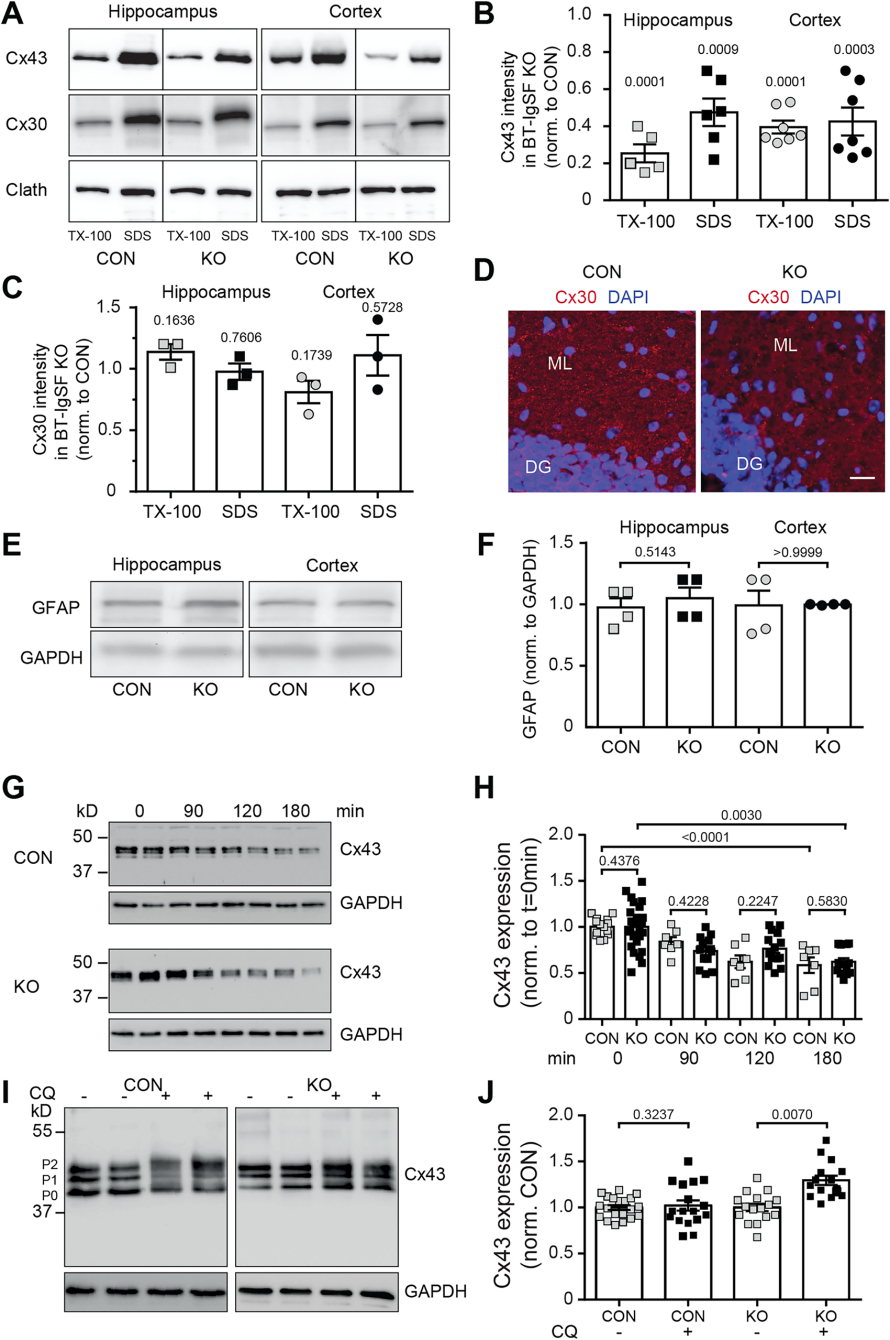
Connexin43 but not connexin30 proteins are reduced in the hippocampus and cortex, and blockers of the protein biosynthesis and proteolysis show increased degradation of connexin43 in the absence of BT-IgSF. ***A***–***C***, Western blots demonstrating reduction of connexin43 in crude membrane fractions from 8-week-old hippocampi or cortices of wild-type and BT-IgSF–deficient mice. The crude membrane fractions were first solubilized in 1% Triton X-100, and unsolubilized material was then solved in 1% SDS; 20 µg of protein was loaded per lane. Loading control is demonstrated by a monoclonal antibody to the heavy chain of clathrin. Quantification of band intensities is shown in ***B***. For the hippocampus, five and six specimens for each genotype were analyzed for Triton X-100 and SDS, respectively; for the cortex, seven and seven specimens for each genotype were analyzed for Triton X-100 and SDS, respectively. In Figure 3*C*, for each condition, three specimens were inspected. Blot intensities of the BT-IgSF mutant were normalized to control (CON) values. *p*-values above the columns indicate significance to controls. Mann–Whitney *U* test. For comparison quantification of Western blots of antibodies to connexin30 are shown in ***C***. ***D***, The localization of connexin30 in the ML of hippocampi is not affected by the absence of BT-IgSF. DG, dentate gyrus; ML, molecular layer. Scale bar, 20 µm. ***E***, ***F***, The expression of GFAP is not reduced in the absence of BT-IgSF. Equal loading is indicated by an antibody to GAPDH. For each condition, four specimens were analyzed. ***G***, ***H***, Astrocytes treated with cycloheximide (100 µg/ml) for times indicated in the figures were lysed (1% SDS in TBS and 1 mM EDTA supplemented with protease blockers) and boiled in SDS sample buffer. Equal amounts were loaded on 10% SDS-PAGE, blotted, and analyzed with rabbit anti-connexin43. Equal loading was controlled by mAb to GAPDH. Quantification of all cycloheximide experiments was done using Image Lab software (Bio-Rad). At each condition, multiple cultures from four mice of each genotype were run on multiple Western blots (7–24). No significance was measured between genotypes; a nested *t* test and an outlier test were applied. ***I***, ***J***, Blocking of lysosomal degradation by CQ (100 µM) for 4 h in astrocyte cultures from control and BT-IgSF knock-outs. At each condition, multiple cultures from three mice of each genotype were run on multiple Western blots (16–20). An outlier test and a nested *t* test were applied, in addition to 3J knock-out data analyzed by Mann–Whitney of the means of the blot replicates for each independent culture (*p* = 0.0286).

To analyze connexin43 in Western blots, we prepared hippocampus and cortex crude membrane fractions from wild types and mutants. Membranes were first treated by Triton X-100 containing buffer followed by SDS of the pellet to separate soluble (primarily nonjunctional) from insoluble (mainly junctional) connexin43. Significant decreases in connexin43 levels in both Triton X-100- and SDS-containing fractions were found in the mutant (Fig. 3*A*,*B*). In contrast to connexin43, connexin30, which is also implicated in the extensive network organization of astrocytes and which can also form gap junction channels together with connexin43 (Willecke et al., 2002; Nagy et al., 2004), is not reduced nor is its overall localization changed in the hippocampus in the absence of BT-IgSF (Fig. 3*C*,*D*). No changes in GFAP protein level were detected (Fig. 3*E*,*F*) in the hippocampus of BT-IgSF mutants. This indicated that the reduction in connexin43 levels was not caused by a decrease in GFAP, that is, a decrease in the number of astrocytes (Fig. 5*K*–*N*).

Connexin43 has a high turnover rate. Therefore, the reduced expression of connexin43 might be caused by decreased biosynthesis, increased degradation by multiple pathways, or by trafficking deficits from the cytoplasm to the plasma membrane (PM; Falk et al., 2016; Laird and Lampe, 2018). We measured connexin43 protein levels using Western blots in a chase experiment after adding the protein synthesis blocker cycloheximide in wild-type and BT-IgSF knock-out astrocyte cultures. In both cultures, the amount of connexin43 protein significantly decreased in the presence of cycloheximide. After 180 min of incubation with cycloheximide, the level of connexin43 protein decreased to 60% of baseline in both genotypes (Fig. 3*G*,*H*). However, no differences between wild-type and knock-out cultures were detected at different incubation periods. Linear regression analysis revealed that 50% of connexin43 would be present after 210.0 min and 235.1 min in wild-type and knock-out astrocytes, respectively (*p* = 0.8869, unpaired *t* test with Welch's correction). Interestingly, the addition of the lysosome inhibitor CQ for 4 h led to an increase in connexin43 protein in knock-out cultures (to 126% on average) but only sparely in control cultures (105%; Fig. 3*I*,*J*) as the change in the latter did not reach statistical significance. These data might indicate that degradation of connexin43 is increased in BT-IgSF knock-out cultures and suggest that BT-IgSF contributes to the stabilization of connexin43. Furthermore, a change in the migration pattern of connexin43 in SDS-PAGE under CQ conditions was detected in astrocytes of both genotypes (Fig. 3*I*). The connexin bands pattern became more diffuse, band P1 appeared weaker, and all bands migrated at a slightly higher position—perhaps indicating increased phosphorylation. The latter shift was stronger in the control. In summary, in the absence of BT-IgSF degradation of connexin43 via the lysosome pathway is increased, which might explain the reduced expression of connexin43 in the hippocampus and cortex of BT-IgSF−/−.

**Figure 4.**
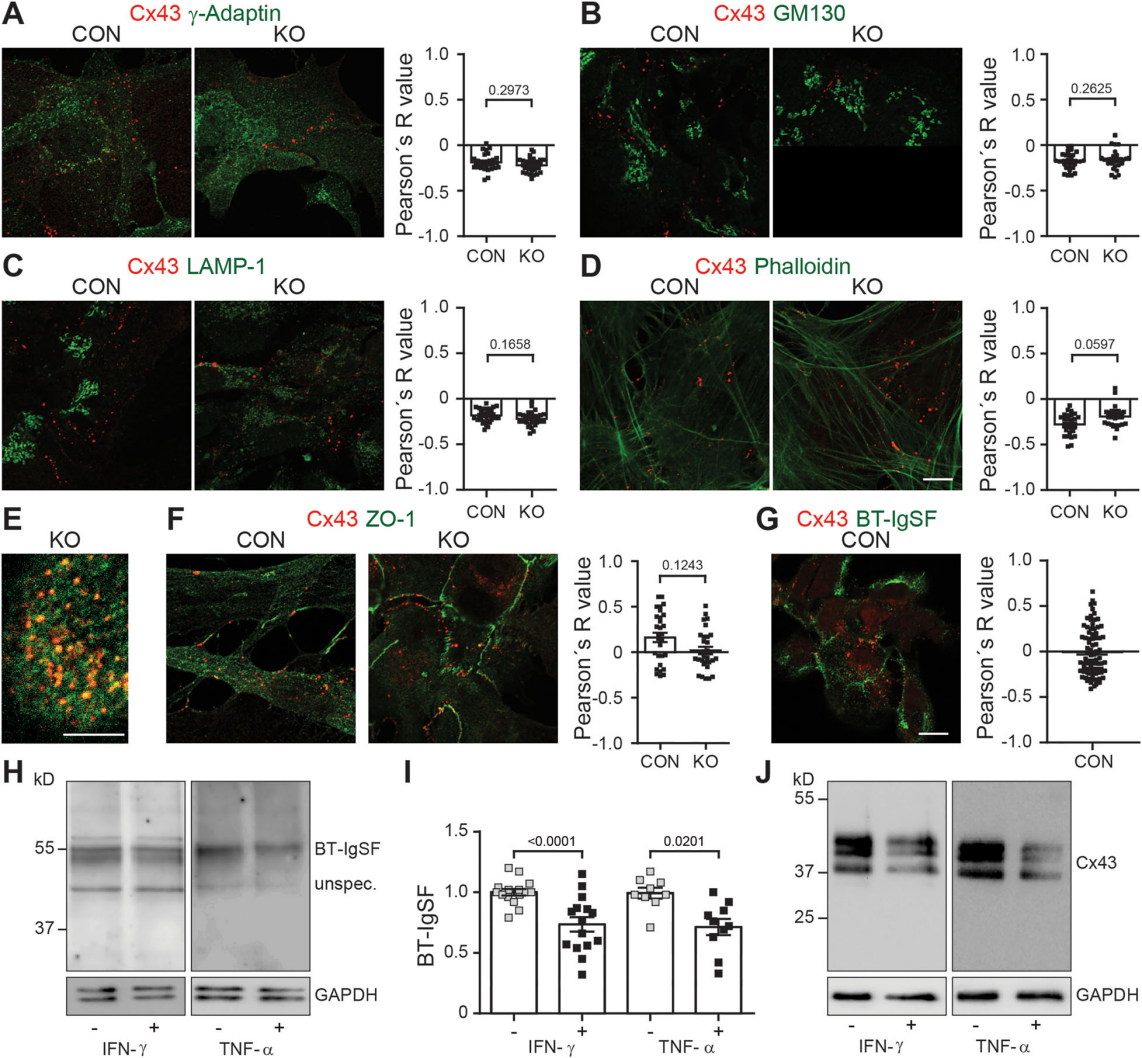
Subcellular localization of connexin43 is not impaired in the absence of BT-IgSF. ***A***–***D***, The subcellular localization of connexin43 in the absence of BT-IgSF and wild-type astrocytes was analyzed by using phalloidin or specific antibodies to intracellular subcellular compartments. Cells from three independent cultures were fixed with 4% paraformaldehyde/PBS (5 min on ice) before application of the antibodies and analyzed as described in Materials and Methods. ***A***, mAb to γ-adaptin and rabbit anti-connexin43, (***B***) mAb to GM130 and rabbit anti-connexin43, (***C***) mAb to LAMP-1 and rabbit anti-connexin43, (***D***) phalloidin-594 and rabbit anti-connexin43. ***E***, Cell surface localization of connexin43 clusters on BT-IgSF–deficient astrocytes in culture. Living cells were incubated with WGA-488 for 45 min on ice and then fixed and permeabilized followed by staining with rabbit anti-connexin43. Connexin43 (red) spots were counted on BT-IgSF−/− astrocytes from three independent cultures (100× objective). Strong colocalization was observed between WGA-488 (green) and connexin43. The numbers are as follows: Experiment 1, 470 connexin43-positive spots (95%) showed colocalization with WGA, 29 not (10 cells analyzed); Experiment 2, 329 (98%) versus 3 (5 cells); and Experiment 3, 519 (98%) versus 14 (7 cells). ***F***, mAb to ZO-1 and rabbit anti-connexin43. ***G***, Rb96 to BT-IgSF and mAb to connexin43. The mean values and standard deviations of the three independent cultures are as follows: −0.02037 (SD 0.2666), 0.07 (SD 0.2768), and −0.0063 (SD 0.2334). The one-way ANOVA value of 0.1632 indicates a similarity between the three cultures. In ***A***–***D*** and ***F*** and in ***G***, *n* = 27–30 and *n* = 82 images were analyzed for the calculation of Pearson's *R*-value, respectively. The number of experimental replicates for ***A***–***G*** was 3. A nested *t* test was applied. Scale bar: ***A–D***, ***F***, ***G***, 10 µm; ***E***, 5 µm. ***H***–***J***, Expression of BT-IgSF protein is decreased in the presence of tumor necrosis factor-α (1 ng/ml) or interferon-γ (10 ng/ml) on astrocytes in culture (***H***, ***I***). Both reagents were applied for 24 h in DMEM without FCS. Four independent wild-type cultures were analyzed in multiple Western blots (IFN-γ, 14 and 15; TNF-α, 9 and 10). For comparison, a reduced expression of connexin43 in the presence of tumor necrosis factor-α or interferon-γ is shown (***J***). Gels using antibodies to BT-IgSF were run under nonreducing conditions. Nested *t* tests were applied and additionally Mann–Whitney *U* test for IFN-γ or TNF-α treatments of the means of biological replicates were performed (*p* = 0.0280 and *p* = 0.0286, respectively).

To test for subcellular localization defects of connexin43 in the absence of BT-IgSF, we compared wild-type and BT-IgSF–deficient astrocytes by staining them with antibodies specific for different compartments including LAMP-1 (lysosomal-associated membrane protein 1), phalloidin (actin cytoskeleton), GM130 (*cis*-Golgi marker), γ-adaptin (secretory vesicles), and ZO-1 (subcompartments such as tight junctions at the cell surface). No increased colocalization between connexin43 and any of these subcellular markers was detected in mutants indicated by a comparison of the Pearson correlation coefficients (Fig. 4*A*–*D*). This might exclude that connexin43 gets retained in an intracellular compartment in BT-IgSF−/− astrocytes. Consequently, connexin43 clusters were found at the cell surface in close association with wheat germ agglutinin that was applied to living astrocyte cultures on ice (Fig. 4*E*). Taken together, these data do not support the notion that the absence of BT-IgSF reduces the expression of connexin43 in astrocytes via impaired intracellular trafficking.

We detected minimal colocalization between connexin43 and the tight junction protein ZO-1 (mean value of Pearson's *R*-value, 0.1631 for wild-type and 0.002 for knock-out) that was further slightly decreased in the BT-IgSF knock-out, possibly due to the fact that connexin43 was present at reduced levels (Fig. 4*F*). In addition, BT-IgSF itself showed very little colocalization with connexin43 (Fig. 4*G*). The occasional colocalization observed could be due to the fact that all of these proteins are present at the PM or might indicate weak and/or transient associations (mean value of Pearson's *R*-value, −0.004568). A direct association between BT-IgSF and connexin43 could not be demonstrated since the available antibodies failed to coprecipitate BT-IgSF and connexin43.

Previously published studies reported that both the tumor necrosis factor-α and interferon-γ reduce the expression of connexin43 on astrocytes and enterocytes, which in turn impairs cell–cell communication (Hinkerohe et al., 2005; Meme et al., 2006; Leaphart et al., 2007; Zhang et al., 2015, 2013). Conversely, BT-IgSF (VSIG3) was found to inhibit the secretion of interferon-γ and tumor necrosis factor-α of activated PBMCs and CD4-positive T cells in cell culture assays (Xie et al., 2021). Therefore, we applied interferon-γ and tumor necrosis factor-α to wild-type astrocyte cultures. Interestingly, the BT-IgSF protein level was diminished in both treatments, which might further strengthen our data on a functional link between BT-IgSF and connexin43 on astrocytes (Fig. 4*H,I*). As expected, connexin43 was also found to be reduced (Fig. 4*J*).

### Reduced astrocyte–astrocyte coupling in the hippocampus and cortex in the absence of BT-IgSF

**Figure 5.**
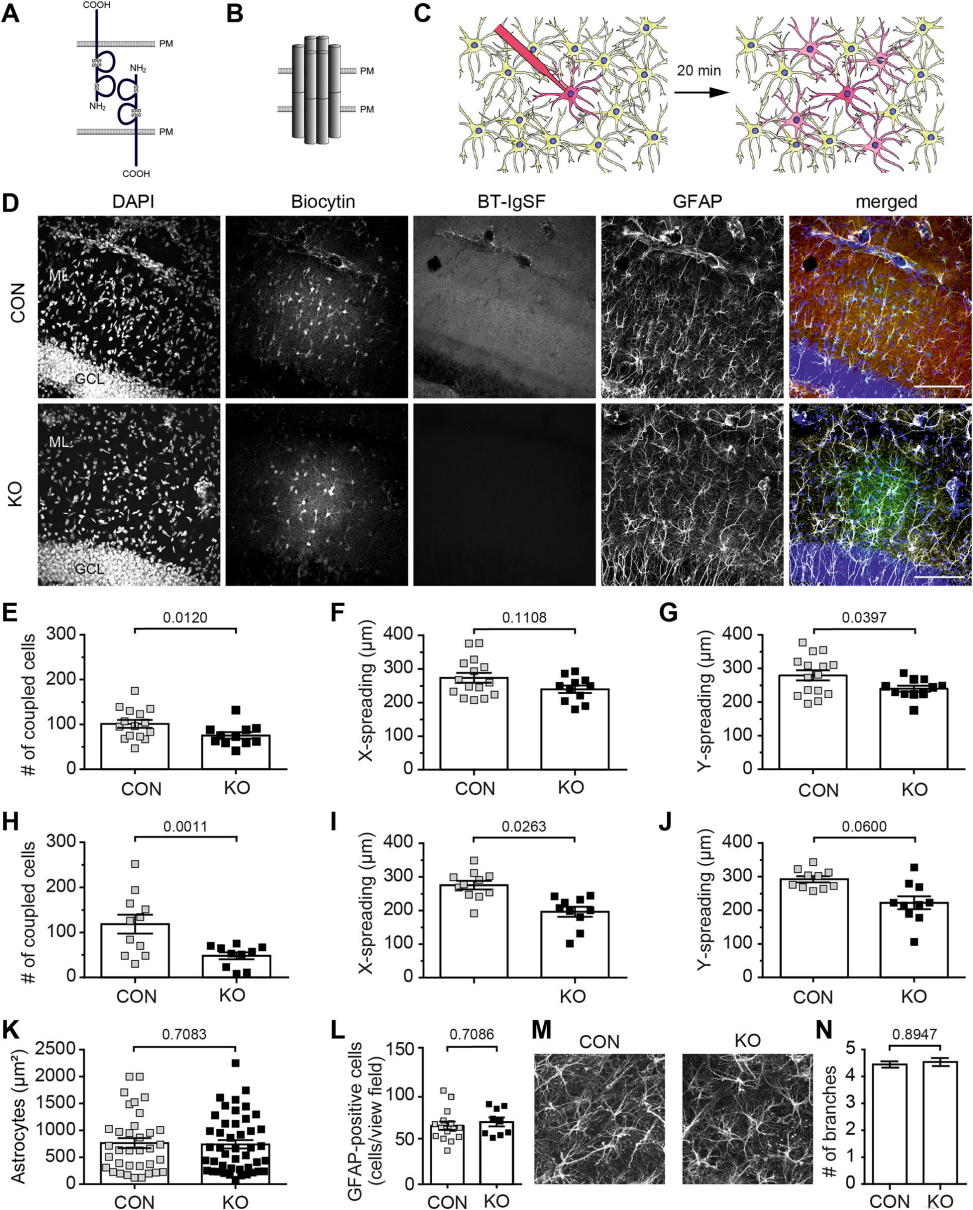
Impaired gap junction–mediated coupling between astrocyte networks in the BT-IgSF knock-out mice (see Extended Data Fig. 5-1 for electrophysiological data). ***A***, Scheme of homophilic binding of BT-IgSF, (***B***) connexin43 coupling, and (***C***) the dye-filling experiment to study gap junctional communication in astrocytes. PM, plasma membrane. ***D***, *z*-stack images of both genotypes of the immunohistochemical analysis for DAPI, biocytin, BT-IgSF, and GFAP staining in the hippocampus. The right images show an overlay of all channels. GCL, granular cell layer; ML, molecular layer. Scale bar, 100 µm. ***E***–***G***, Scatter plots showing the network size (***E***, number of coupled cells), tracer spread alongside (***F***, tracer spread *x*), or tracer spread perpendicular to the Schaffer collaterals (***G***, tracer spread *y*). A total of 15 dye-filled slices from three control mice and 11 slices from four BT-IgSF knock-out mice were analyzed. On average, 101 ± 8.851 (mean ± SD) biocytin-positive hippocampal astrocytes were coupled in the wild-type, whereas in the knock-out, only 78 ± 7.837 (mean ± SD) biocytin-positive astrocytes were found to form a network. Nested *t* test. ***H***–***J***, Scatter blots of dye coupling of astrocytes in the cortex. On average, 118 ± 20.95 (mean ± SD) astrocytes in control and 48 ± 7.86 (mean ± SD) in the BT-IgSF knock-out form a network (11 dye-filled slices from four controls and 10 slices from four knock-outs). Nested *t* test. ***K***, The area of individual cultured astrocytes is not affected by the absence of BT-IgSF (three independent cultures of each genotype). Nested *t* test. ***L***, The number of GFAP-positive astrocytes is not reduced in the absence of BT-IgSF. GFAP-positive cells were counted in microscopic view fields (350 µm × 350 µm) in the ML of sections of hippocampi from wild-type and BT-IgSF knock-out mice. A total of 964 cells in 15 view fields and 616 cells in 9 view fields were counted for wild-type and knock-out, respectively. The numbers of replicates were 4 for the control and 3 for the BT-IgSF knock-out. Nested *t* test and in addition Mann–Whitney of the means of the replicates (*p* = 0.700). ***M***, Images of GFAP-positive astrocytes in the ML of the hippocampus from wild-type and BT-IgSF–deficient mice indicating a similar morphology. ***N***, Branching of GFAP-positive cells remains unchanged in the absence of BT-IgSF. Primary GFAP-positive branches were counted in the ML of the hippocampus. In total, 46 and 41 cells were analyzed for wild-type and knock-out, respectively. The numbers of replicates were 4 for the control and 3 for the BT-IgSF knock-out. Nested *t* test.

A typical feature of astrocytes in the brain is their organization in vast networks that communicate with one another via gap junction channels formed by connexins (Giaume et al., 2010). In order to investigate the effect of BT-IgSF ablation on astrocytic network size in the hippocampus and cortex, we performed dye-coupling experiments (see schemes shown in Fig. 5*A*–*C*). Astrocytes were identified in acutely isolated mouse brain slices by SR-101 that specifically labels astroglia (Nimmerjahn et al., 2004). Individual astrocytes in the hippocampus ML or cortical layers II–IV from horizontal slices were dye-loaded with biocytin via a patch pipette. Diffusion of the dye throughout the astrocytic network allows for the visualization and quantification of the extent of gap junction–mediated cell–cell coupling (Fig. 5*D*).

10.1523/ENEURO.0283-23.2024.f5-1 Figure 5-1 **Analysis of dye spreading in astrocytes** A) Bright field and fluorescence images of a patch-clamped astrocyte in the hippocampus. *Top* Sulforhodamine (SR101) staining of the patched astrocyte. The patch-clamp pipettes outline is marked as a dotted line. *Middle* Bright field (BF) image of the field of interest. The arrow marks the position of the patched astrocyte. *Bottom* To ensure success of the dialysis, Lucifer yellow (LY) was added to the pipette-solution. Bar, 20µm. B) Typical current profiles of an astrocyte in the wildtype (top) and knockout group (bottom) clamped at -70 mV in response to 10 de- and hyperpolarizing voltage-steps in hippocampus. Only cells which displayed a series resistance of ≤ 125% of the initial value after 20 min of dialysis were included in the analysis. C) The graph shows the averaged current to voltage relationship of both genotypes at the start and the end of the 20 min dialysis period in hippocampus (black: wildtype, grey: BT-IgSF knockout). D and E) The boxplot graphs compare the membrane capacitance and membrane resistance of hippocampal astrocytes at the start and the end of the dialysis. No significant differences were observed in either comparison (One-way ANOVA, p > 0.05). Data in C to E were compiled from dye spreading measurements. Similar results on the current profiles, membrane capacitance and membrane resistance were also obtained from cortical astrocytes. Download Figure 5-1, TIF file.

In the hippocampus as well as in the cortex, the number of coupled astrocytes per injected cell was significantly decreased in mutant animals. A total of 15 dye-filled slices from three control mice and 11 slices from four BT-IgSF knock-out mice were used for immunohistochemical analysis of hippocampal coupled astrocytic networks. On average, 101 ± 8.851 (mean ± SD) biocytin-positive hippocampal astrocytes were coupled in the wild-type, whereas in the knock-out, only 78 ± 7.837 (mean ± SD) biocytin-positive astrocytes were found to form a network (Fig. 4*E*–*G*). In the cortex, the differences between wild-type and knock-out were even more pronounced: on average, 118 ± 20.95 (mean ± SD) astrocytes in control and only 48 ± 7.86 (mean ± SD) in the BT-IgSF knock-out form a network (11 dye-filled slices from four controls and 10 slices from four knock-outs; Fig. 5*H*–*J*). Extended Data Figure 5-1 illustrates the passive membrane properties of hippocampal astrocytes in both groups over the course of the dialysis period. We observed no significant differences in the current–voltage relationship, membrane resistance, or membrane capacitance (*p* > 0.05) between genotypes or between the start and the end of the dialysis period, indicating that these factors were not responsible for the change in the extent of the astrocytic network. Similar results were obtained for passive membrane properties in cortical astrocytes. Further, neither the density of GFAP-positive astrocytes nor their complexity (arborization) was reduced in the absence of BT-IgSF, suggesting that altered morphological parameters of astrocytes are unlikely to cause reduced cell–cell coupling in the absence of BT-IgSF (Fig. 5*K*–*N*).

In summary, we detected reduced astrocyte–astrocyte coupling in the hippocampus and the cortex in the absence of BT-IgSF. We assign this to the observed changes in connexin43 expression, that is, the reduction of the connexin43 protein levels and/or its altered clustered localization.

### The absence of BT-IgSF disrupts the localization of connexin43 on ependymal cells

**Figure 6.**
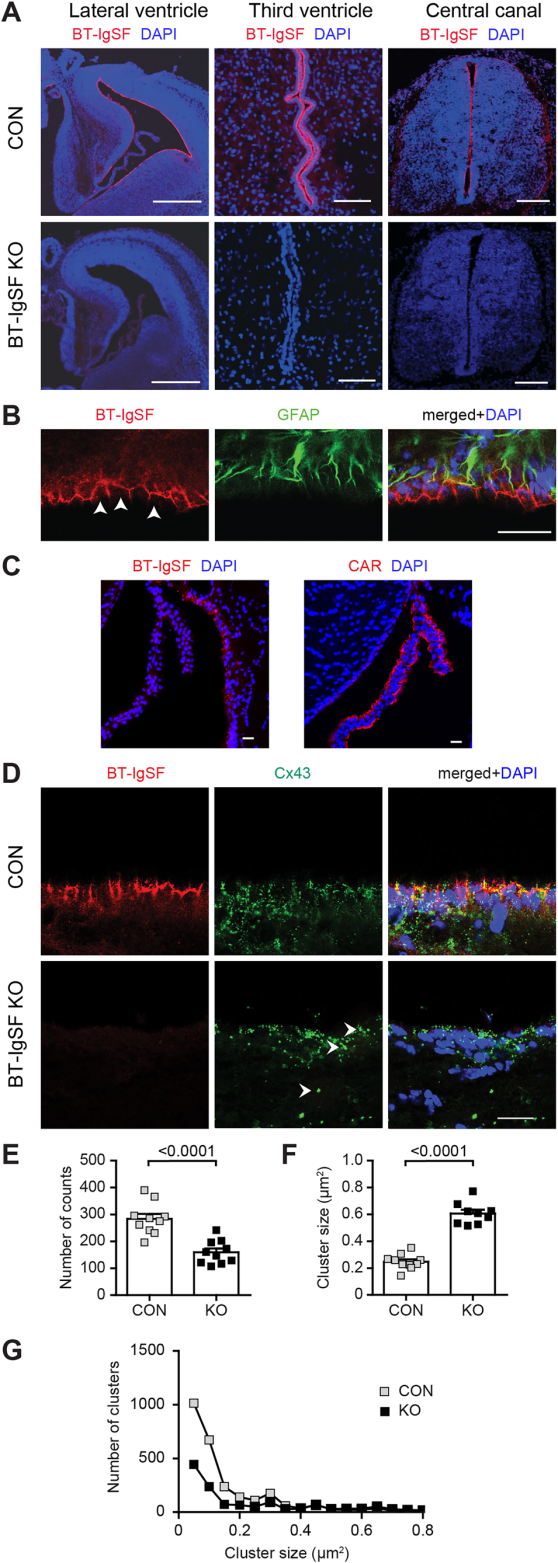
Expression of BT-IgSF on ependymal cells and impaired localization of connexin43 on ependymal cells in the absence of BT-IgSF (see Extended Data Fig. 6-1 for BT-IgSF antibody specificity on ependymal cells). ***A***, Expression of BT-IgSF in ependymal cells of the lateral, third ventricle, and central canal by immunohistochemistry using an antibody to the extracellular domain of BT-IgSF. E15 lateral ventricle: scale bar, 400 µm; P7 third ventricle: scale bar, 50 µm; central canal of an E12.5 spinal cord, transversal section; dorsal is up, scale bar, 100 µm. Red, anti–BT-IgSF; blue, DAPI. For the specificity of the antibody, sections of BT-IgSF knock-out tissue are shown. ***B***, Higher magnification of the ependymal cell layer showing expression of BT-IgSF at lateral and basal sides of ependymal cells in the lateral ventricle of an adult wild-type mouse. Colocalization of BT-IgSF and GFAP in the ependyma is shown. The ventricle and the apical side of the ependymal cells (indicated by arrowheads) are at the bottom of the image. Scale bar, 20 µm. ***C***, BT-IgSF is not or only weakly found at the choroid plexus in contrast to the related CAR. Coronal sections from regions of the lateral ventricle from a 19-week-old mouse were stained with rabbit antibodies to BT-IgSF (Rb95) or CAR (Rb80) and DAPI. ***D***, Clustering of connexin43 on ependymal cells of the lateral ventricle from adult wild-type and BT-IgSF knock-out mice. The ventricle is at the top of the images. The arrowheads indicate a large connexin43 cluster. Scale bar, 20 µm. ***E***–***G***, Quantification of the number of connexin43 spots, size, and distribution of number versus size as described for astrocytes. Control, 283.5 ± 58.8; BT-IgSF−/−, 132.9 ± 145.8 (mean ± SD) spots from 10 view fields (63× objective) from three animals per genotype. Nested *t* test.

Ependymal cells are specialized multiciliated glial cells that line the ventricles and form an interface between the cerebrospinal fluid (CSF) and brain parenchyma (Deng et al., 2023). These cells also express GFAP (Roessmann et al., 1980). Ependymal cells contact each other via connexin43 containing gap junctions and are implicated in barrier formation as well as the production and circulation of CSF (Saunders et al., 2018). To ask whether the absence of BT-IgSF also affects the localization of connexin43 on ependymal cells, we characterized the expression of BT-IgSF in the brain by immunohistochemistry. We detected a strong localization of BT-IgSF on cells lining all brain ventricles at embryonic as well as postnatal stages but only very weak staining in the neural tissue directly adjacent to the ependymal cell layer (Fig. 6*A*, see also Extended Data Fig. 6-1 for antibody specificity). Higher magnifications showed that BT-IgSF was found at the lateral and basal surfaces of ependymal cells, but not at the apical side that faces the ventricle (Fig. 6*B*). The choroid plexus that expresses the related IgCAM CAR stained weakly for BT-IgSF (Fig. 6*C*). Matching the deficits that we found in astrocytes, we also found a decreased number of spots and an increased clustering of connexin43 in BT-IgSF mutant ependymal cells compared with wild types (Fig. 6*D*–*G*).

10.1523/ENEURO.0283-23.2024.f6-1 Figure 6-1 **Localization of BT-IgSF at the lateral ventricle at P14 and P80.** A and B) Coronal sections of the brain at P14 and P80 were stained with rabbit anti-BT-IgSF. BT-IgSF is strongly localized in cells lining the lateral ventricle. The dashed boxes indicate the position shown in row two or four. Absence of staining in BT-IgSF knockout tissues demonstrates specificity of antibodies to BT-IgSF. LV, lateral ventricle. Scale bar, first and third row, 1 mm; scale bar second and fourth row, 20 µm. Download Figure 6-1, TIF file.

### The localization of connexin36 in neurons is not affected by the absence of BT-IgSF

**Figure 7.**
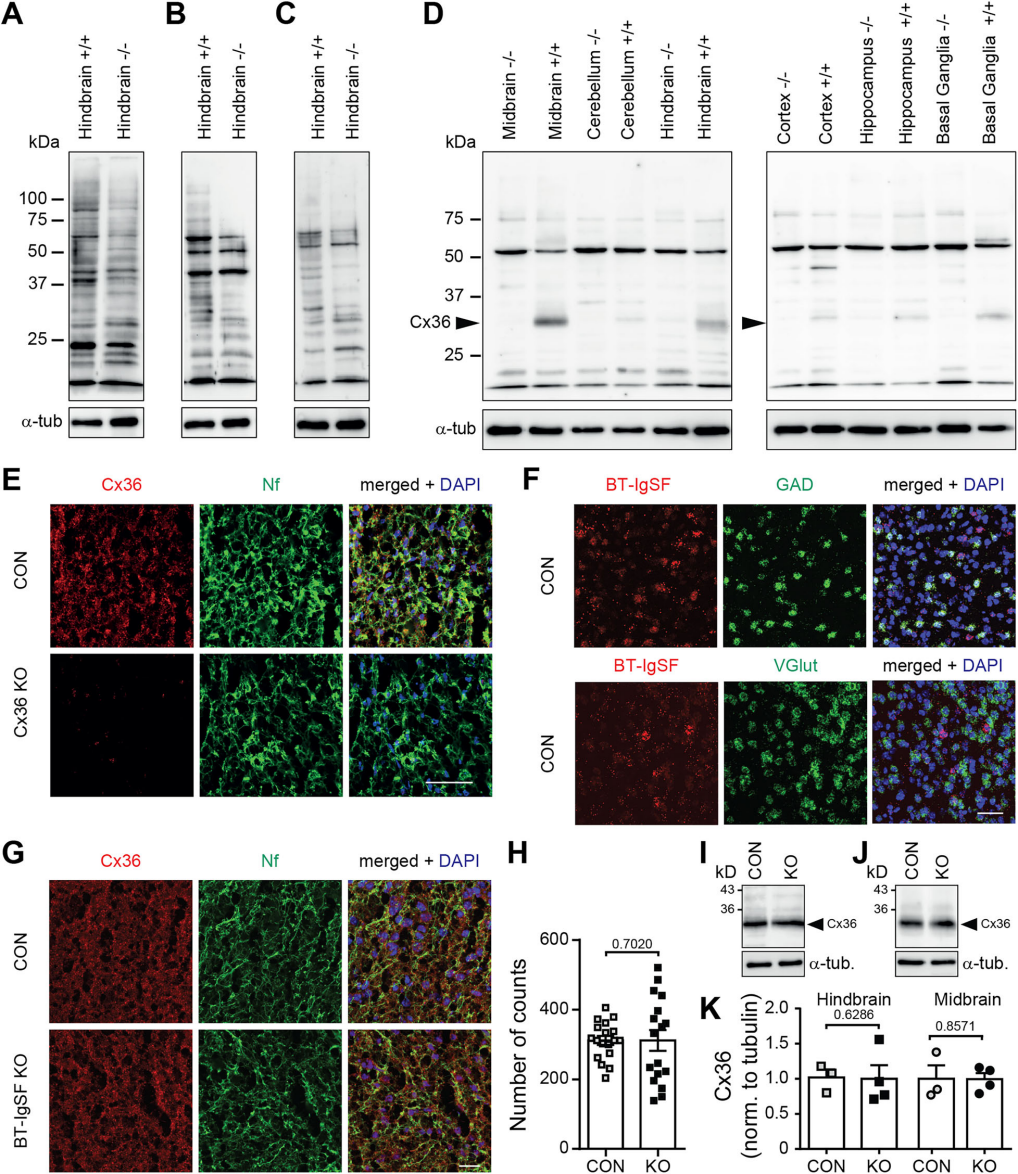
Connexin36 localization on neurons is not impaired in the absence of BT-IgSF. ***A***–***D***, Specificity of antibodies to connexin36 in Western blots using crude membrane fractions from wild-type and connexin36-deficient P10 brain tissues. In addition, 12 µg of protein was loaded per lane. In ***A***, mAb to connexin36 (sc-398063); in ***B*,** mAb to connexin36 (8F6.2); in ***C*,** rabbit to connexin36 (364600); and in ***D*,** rabbit antibody to connexin36 generated in this study (amino acid residues 99–197) are shown. Satisfactory specificity could only be demonstrated for the antibody shown in ***D***. A 34 kD protein (arrowheads) was specifically detected in wild-type but not in connexin36-deficient tissues. However, a prominent unspecific band at 52 kD was detected in both genotypes. ***E***, Specificity of rabbit antibody to connexin36 (residues 99–197) in sagittal sections of P10 superior colliculus from wild-type and connexin36-deficient mice. Nf, neurofilament. ***F***, RNAscope demonstrating neuronal expression of BT-IgSF in sagittal sections from P10 superior colliculus. ***G***, ***H***, Localization and clustering of connexin36 are not altered in P10 sagittal sections of the midbrain in the absence of BT-IgSF. Scale bar, 20 µm. Nf, neurofilament. The numbers of connexin36 spots in the midbrain were counted in three cryosections of four animals of each genotype. In both cases, 2,500 µm^2^ were analyzed by ImageJ software for each section. Nested *t* test. ***I***–***K***, Connexin36 protein is not altered in the absence of BT-IgSF. Hindbrain (***I***) and midbrain (***J***), control *n* = 3; knock-out, *n* = 4. Mann–Whitney *U* test.

In the brain, connexin43 is restricted to astrocytes, whereas connexin36 is the main connexin in neurons and is primarily expressed during embryonic and early postnatal developmental stages (Condorelli et al., 1998; Söhl et al., 1998; Gulisano et al., 2000; Degen et al., 2004; Rubio and Nagy, 2015). We, therefore, asked whether the absence of BT-IgSF also affects the localization of the gap junction protein connexin36 in neurons. Commercially available antibodies to connexin36 were of limited use in our hands, and we therefore generated a polyclonal antibody to the cytoplasmic stretch (residues 99–197) of mouse connexin36 (Fig. 7*A*–*D*). Although this antibody also showed some unspecific binding in Western blots, it clearly labeled connexin36 at a molecular mass of 34 kDa in wild-type but not in connexin36-deficient neural tissues (Fig. 7*D*). The specificity of this antibody to connexin36 could also be demonstrated in sections of wild-type or connexin36-deficient brain tissue (Fig. 7*E*). The strongest expression of connexin36 was detected in the midbrain and hindbrain at postnatal day 10, while much weaker expression levels were found in the cerebellum, basal ganglia, hippocampus, and cortex (Fig. 7*D*). We used single-molecule fluorescent in situ hybridization (using RNAscope) to define the neuronal cell types expressing *Bt-igsf* mRNAs and observed coexpression with *vGlut1* and *GAD65* in the midbrain, indicating that *Bt-igsf* is expressed in both excitatory and inhibitory neurons (Fig. 7*F*). Similar expression patterns were seen in the hippocampus (Extended Data Fig. 2-2). Since the strongest expression of connexin36 was detected in the midbrain, we analyzed this region in more depth. No differences in the localization or expression of connexin36 protein were detected in the absence of BT-IgSF in the midbrain and hindbrain (Fig. 7*G*–*K*). We conclude that BT-IgSF modulates the expression of connexin43 specifically in astrocytes and ependymal cells, but does not affect localization or clustering of connexin36 in neurons.

## Discussion

Here, we show that the Ig cell adhesion molecule BT-IgSF (IgSF11/VSIG-3) is highly expressed in astrocytes and ependymal cells of the mouse brain. Our functional analysis demonstrates that BT-IgSF is essential for the correct expression and subcellular localization of connexin43 in astrocytes and ependymal cells. In the absence of BT-IgSF, the level of connexin43 protein is severely reduced and fewer, but larger clusters of connexin43 are formed on the surface of astrocytes and ependymal cells in the brain. Importantly, our analysis of gap junction coupling in BT-IgSF knock-outs revealed a reduced astrocytic network in the cortex and hippocampus. However, this impaired coupling was not due to morphological changes of astrocytes in the absence of BT-IgSF. Astrocytes generate a complex syncytial network allowing them to interact with many neighboring cells to control a number of physiological processes like neurotransmission, and disruptions of gap junction coupling are found in a broad spectrum of diseases such as neurodegeneration or epilepsy (Mayorquin et al., 2018; Huang et al., 2021; Mazaud et al., 2021; Bedner and Steinhäuser, 2023). A change in the localization pattern or expression level of connexin36 in neurons was not detected in the absence of BT-IgSF.

BT-IgSF is a member of a small and evolutionarily conserved subfamily of IgCAMs of which CAR was the founding member. This set of proteins mediates homotypic cell adhesion and shares a common overall extracellular domain structure and a highly related amino acid sequence. A number of studies that relied on mouse mutants suggested that these proteins might undertake similar functions as they help to organize or regulate gap junctions in a variety of cell types (Falk, 2020; Rathjen, 2020). For example, in the absence of CAR, connexin43 and connexin45 are reduced in the heart, resulting in impaired electrical conduction at the atrioventricular node (Lim et al., 2008; Lisewski et al., 2008; Pazirandeh et al., 2011). In addition, cultured embryonic CAR-deficient cardiomyocytes showed increased calcium cycling, increased beating frequency, altered connexin43 clustering, and impaired dye coupling (Matthaeus et al., 2022). Another example is CLMP as the absence of this adhesion protein impaired the function of smooth muscle cells of the intestine and ureter. Accordingly, uncoordinated calcium signaling provoked a disturbed contraction of the intestine and ureter. Notably, the level of connexin43 and connexin45 proteins, but not their mRNAs, was severely reduced in the smooth muscle layers (Langhorst et al., 2018; Rathjen and Jüttner, 2023). Our studies on BT-IgSF described here provide an additional example of the way this subgroup of proteins plays a part in gap junction–mediated cell–cell communication. The absence of BT-IgSF also affects the expression and localization pattern of connexin43 in astrocytes and ependymal cells. This is consistent with previously published data on the mislocalization of connexin43 in the Sertoli cells of the testes, which leads to infertility and the functional impairment of the blood–testis barrier (Pelz et al., 2017). Thus, all three members of the family, BT-IgSF, CLMP, and CAR, are required for appropriate gap junction–mediated cell–cell communication.

### How might BT-IgSF exert its function on connexin43 in astrocytes?

Connexin43, much like other connexins, has a dynamic “life cycle” and possesses a half-life time of ∼1–5 h. Furthermore, connexins have a complex biosynthesis and can be degraded by multiple pathways. Many proteins are known to interact with connexins during all stages of the life cycle of a gap junction (Falk et al., 2016; Solan and Lampe, 2016). The reduced expression of connexin43 in BT-IgSF knock-out astrocytes appears to be caused by increased degradation as revealed by our lysosomal pathway inhibition experiments and might indicate a stabilization function of BT-IgSF on connexin43 at gap junctions. However, the intracellular localization of connexin43 to specific compartments was not altered, suggesting that intracellular trafficking of connexin43 might not be disturbed in the absence of BT-IgSF. The decreased amount of connexin43 might result in increased clustering at the cell surface and might therefore be a secondary effect. Interestingly, treatment of astrocyte cultures with tumor necrosis factor-α or interferon-γ caused a reduction of BT-IgSF that parallels the decrease of connexin43 by these factors. The latter finding was previously described in enterocytes of the intestine and astrocytes (Hinkerohe et al., 2005; Meme et al., 2006; Leaphart et al., 2007; Zhang et al., 2015, 2013). Therefore, cytokines might be players that regulate the expression of BT-IgSF protein that in turn regulates connexin43 expression or localization. Reduced astrocyte–astrocyte coupling was also recently described in knockdown experiments of the Ig cell adhesion molecule HepaCAM (also termed GlialCAM) that, at the structural level, is only distantly related to CAR subgroup members (Moh et al., 2005; Favre-Kontula et al., 2008). The authors found a reduced morphological complexity, including arborization of astrocytic protrusions, and a reduction in the territory covered by astrocytes. In addition, increased clustering of connexin43 was found in HepaCAM mutants and was accompanied by decreased dye coupling (Baldwin et al., 2021). Except for the increased clustering of connexin43, we neither detected similar morphological changes in astrocytes of BT-IgSF knock-outs nor found a reduction in astrocyte cell density in the ML of the hippocampus. Moreover, HepaCAM is strongly expressed in the white matter, implicated in leukodystrophy and regulates the activity of glioblastoma cells (Favre-Kontula et al., 2008; López-Hernández et al., 2011; Jeworutzki et al., 2012; De et al., 2023), whereas the overall structure of the brain appears not to be affected by the absence of BT-IgSF (Figs. 1*B*,*D*, 6*A*, Extended Data Figs. 2-1, 6-1). Therefore, HepaCAM and BT-IgSF seemingly play different roles, although in both cases their mutation resulted in impaired coupling. Furthermore, biochemical experiments established the direct association of HepaCAM with connexin43 at the cell surface (Wu et al., 2016; Baldwin et al., 2021). By contrast, based on our colocalization studies in cultured astrocytes, we only occasionally detected BT-IgSF in close proximity with connexin43 at the cell surface of astrocytes, and we could not detect a direct interaction between the two proteins in coprecipitation experiments.

Colocalization of BT-IgSF with the scaffolding protein ZO-1 (zona occludens-1) at the cell surface was detected in the Sertoli cells in confocal images (Pelz et al., 2017). BT-IgSF harbors a PDZ-binding motif at its C-terminal domain and scaffolding proteins such as ZO-1 bind via a PDZ domain to most connexins (Hervé et al., 2014). Therefore, one might speculate that ZO-1 binds to BT-IgSF via its PDZ domain1 and to connexin43 via its PDZ domain 2, thus regulating connexin43 localization (Duffy et al., 2002). Furthermore, ZO-1 has been shown to control gap junction assembly as well as localization and influence plaque size in cell cultures (Hunter et al., 2005; Laing et al., 2005; Rhett et al., 2011). A frameshift mutation in the connexin43 gene in human patients suffering from oculodentodigital dysplasia disrupts the connexin43–ZO-1 interaction (van Steensel et al., 2005; De Bock et al., 2013). Despite these findings in other cell types, colocalization of ZO-1 with connexin43 or colocalization of BT-IgSF with connexin43 was only rarely detected at the PM of astrocytes. Nevertheless, a transient association of these components cannot be excluded. Additional studies with mice expressing mutant versions of BT-IgSF and ZO-1 to investigate possible interactions between BT-IgSF, ZO-1, and connexin43 might clarify whether these complexes play a role in connexin43 assembly or in stabilizing connexin43 at the PM of astrocytes. Currently, the question of precisely how BT-IgSF controls connexin43 localization and expression needs to be investigated further. Additional studies are needed to thoroughly understand a possible signal transduction pathway downstream of BT-IgSF that modulates the de novo incorporation or removal of connexin43 to or from the PM.

### BT-IgSF might have different functions in neurons and glial cells

Previously published knockdown studies using cultured hippocampal neurons from the CA1 region implicated BT-IgSF (IgSF11) in synaptic transmission through interactions with PSD95 and AMPA receptors (Jang et al., 2015). In these in vitro experiments, the BT-IgSF (IgSF11) knockdown caused increased mobility and endocytosis of AMPA receptors, suggesting that BT-IgSF is important for the stabilization of AMPA receptors in the neuronal PM. In accordance, BT-IgSF–deficient mice revealed a moderately decreased excitatory synaptic strength in the DG and enhanced long-term potentiation in CA1 (Jang et al., 2015). Furthermore, BT-IgSF (IgSF11) was found to regulate the innervation of axons of chandelier cells on initial axon segments of pyramidal neurons (Hayano et al., 2021). To verify if our findings also apply to connexin localization in neurons, we extended our study and analyzed localization and expression of connexin36, the main neuronal connexin (Condorelli et al., 1998; Söhl et al., 1998; Gulisano et al., 2000; Degen et al., 2004; Rubio and Nagy, 2015). No differences in the localization or expression of connexin36 protein were detected in the absence of BT-IgSF in the midbrain. We concluded that BT-IgSF might have a different function in astrocytes and neurons, specifically modulating the expression of connexin43 in astrocytes and ependymal cells, but not affecting the distribution of connexin36 in neurons.

### What might be the consequences of astrocyte network disturbance for neuronal function?

Astrocytes form an elaborate network to control a number of physiological processes in the brain. Disrupted communication of astrocytes in the absence of BT-IgSF could interfere with the coordination of astrocytic calcium waves or might affect synaptic activity. The close contact of astrocytic branches with synapses allows astrocytes to sense neuronal activity via their ion channels and neurotransmitter receptors (Ventura and Harris, 1999). Disruption of astrocytic networks, for example, by inactivation of connexin43 and connexin30 reduced synaptic transmission (Perea et al., 2009; Giaume et al., 2010; Pannasch et al., 2011; Pereda, 2014; Charvériat et al., 2017; Hardy et al., 2021). Accordingly, connexin30 and astrocyte-targeted connexin43 knock-out mice as well as connexin43 and connexin30 double knock-out mice display impaired performance in sensorimotor and spatial memory tasks (Theis et al., 2003; Lutz et al., 2009). Astrocyte coupling is also altered in epilepsy (Steinhäuser and Boison, 2012; Bedner and Steinhäuser, 2023). Decreased coupling among astrocytes promotes neuronal hyperexcitability and attenuates seizure-induced histopathological outcomes (Deshpande et al., 2020). Astrocytic dysfunction is implicated in a number of neurodevelopmental disorders (Molofsk et al., 2012; Tan et al., 2021). Whether the behavioral deficits observed in BT-IgSF knock-outs are caused by reduced astrocyte–astrocyte coupling or by deficits in the AMPA receptor trafficking needs further investigation using astrocyte- or neuron-specific ablation of BT-IgSF (Montag et al., 2023).

In the brain and spinal cord, ependymal cells line the ventricles and bear multiple cilia that beat in a concerted manner at their apical surface to drive CSF circulation (Spassky and Meunier, 2017). This unidirectional movement might be coordinated by gap junction–mediated cell–cell communication. Consistent with this hypothesis, zebrafish embryos injected with connexin43 morpholinos and connexin43-deficient mouse embryos exhibit a decreased number of cilia as well as diminished beating (Zhang et al., 2020).

In conclusion, in the present study, we identified BT-IgSF as a crucial molecular helper to establish the correct localization of connexin43 on astrocytes and ependymal cells. In astrocytes, this is essential for effective cell–cell coupling. Whether BT-IgSF's selective absence in astrocytes or ependymal cells is also important for neurotransmission or the coordinated movement of cilia, respectively, should be investigated in future work.

## Synthesis

Reviewing Editor: Lynda Erskine, University of Aberdeen Decisions are customarily a result of the Reviewing Editor and the peer reviewers coming together and discussing their recommendations until a consensus is reached. When revisions are invited, a fact-based synthesis statement explaining their decision and outlining what is needed to prepare a revision will be listed below. The following reviewer(s) agreed to reveal their identity: NONE.

Apologies for the time it has taken to get this decision to you. This was due to difficulties in securing reviewers for the manuscript. Below is a synthesis of the reviews outlining the concerns the reviewers and I would like you to consider in preparing a revised manuscript. These should all be able to be addressed through text changes. Apologies again for the time the review process has taken.

General comments:

In this manuscript, the authors report the critical role for BT-IgSF in regulating Connexin 43 expression, localization, and clustering in both astrocytes and ependymal cells. The bulk of the work in the manuscript centers on astrocytes, where the authors find defects in Connexin 43 localization both in vitro and in vivo, and identify enhanced lysosomal degradation of Connexin 43 in the absence of BT-IgSF. Importantly, the authors demonstrate that loss of BT-IgSF impairs astrocyte coupling in both the cortex and hippocampus. The identification of novel regulators of Connexin 43 function in astrocytes is exciting and important, given the many critical functions of Connexin 43 both in astrocytes and in other tissues in the body (e.g., heart). However, there are concerns about some data interpretation and there is a lack of reporting of sample size and statistical methods throughout the paper.

1. Both the title and the abstract state the BT-IgSF is important for ependymal cell-cell coupling, but nowhere in the manuscript is this shown or tested. The authors show impacts to Cx43 localization in ependymal cells, but do not determine whether or not this impacts the coupling. It is suggested that the authors remove these statements from the title and abstract as well as the speculation in the results that ependymal cell-cell coupling could be affected (or find a way to study ependymal cell coupling).

2. Some brief information is given in the figure legends on statistical methods. However, no information is provided on the methods used to determine if the data is distributed normally or the specific test used in each experiment. See below for further comments on reporting of sample size and statistical methods.

2. Figure 1: Sample size information is missing from the legend, as is statistical test information. What does each data point represent in 1E, F, H, and I? How many separate experiments were performed?

3. The authors state that the results from Figure 1 "indicate that BT-IgSF is essential for the correctly localization of connexin 43 at the plasma membrane." However, no data is provided with regards to presence of Cx43 at the plasma membrane, as no plasma membrane marker is used in the KO samples. The authors should either repeat these experiments including a membrane marker in all conditions and a quantification of the percentage of Cx43 signal at the plasma membrane, or change the text to remove the reference to the plasma membrane.

4. Figure 3: Sample size information and statistical test information is missing from the figure legend. The authors report that connexin 30 clustering is not changed in KO mice, but there is no quantitative data to support this conclusion.

5. The conclusion that the authors make from Figure 3I and 3J is that Cx43 lysosomal degradation is increased in the absence of BT-IgSF. However, this finding in context of the results from Figure G and H, which shows similar rate of Cx43 degradation. Is there another type of degradation for Cx43 that is predominant in the WT cultures? This should be discussed in the text.

It would be helpful to include some information and citations demonstrating the specificity of chloroquine for the lysosomal degradation pathway.

6. Figure 4: Quantification is missing for panel E. 4J is discussed in the text before 4H and 4I, so perhaps the panels should be rearranged? In the figure legends, the number of images analyzed is mentioned, but it is unclear how many experimental replicates were used. Was this all from the same experiment? Were multiple experiments performed multiple images quantified from each experiment?

Additionally, it is hard to make out any detail in the images shown. Higher magnification images may help.

7. Figure 5: in the legend, L and N state number of cells counted, but do not indicate the number of biological replicates (i.e., mice) that were used.

8. Figure 6: it would be nice to include arrows and/or labels in panel B to denote the apical and basal sides of the ependymal cell layer. Does GFAP label the ependymal cells? Once again, no information provided on sample size, replicates, or statistical tests.

9. Figure 7: There is no quantitative data presented along with panel G to support the conclusion that Cx36 localization/clustering is not altered in the KOs.

10. There are a number of typographical errors throughout the manuscript.
