# Supplementary figures and images for: The IgCAM BT-IgSF (IgSF11) Is Essential for Connexin43-Mediated Astrocyte–Astrocyte Coupling in Mice
Source: eNeuro. 2024 Mar 6;11(3):ENEURO.0283-23.2024. doi: 10.1523/ENEURO.0283-23.2024 (PMC10957231; doi:10.1523/ENEURO.0283-23.2024)

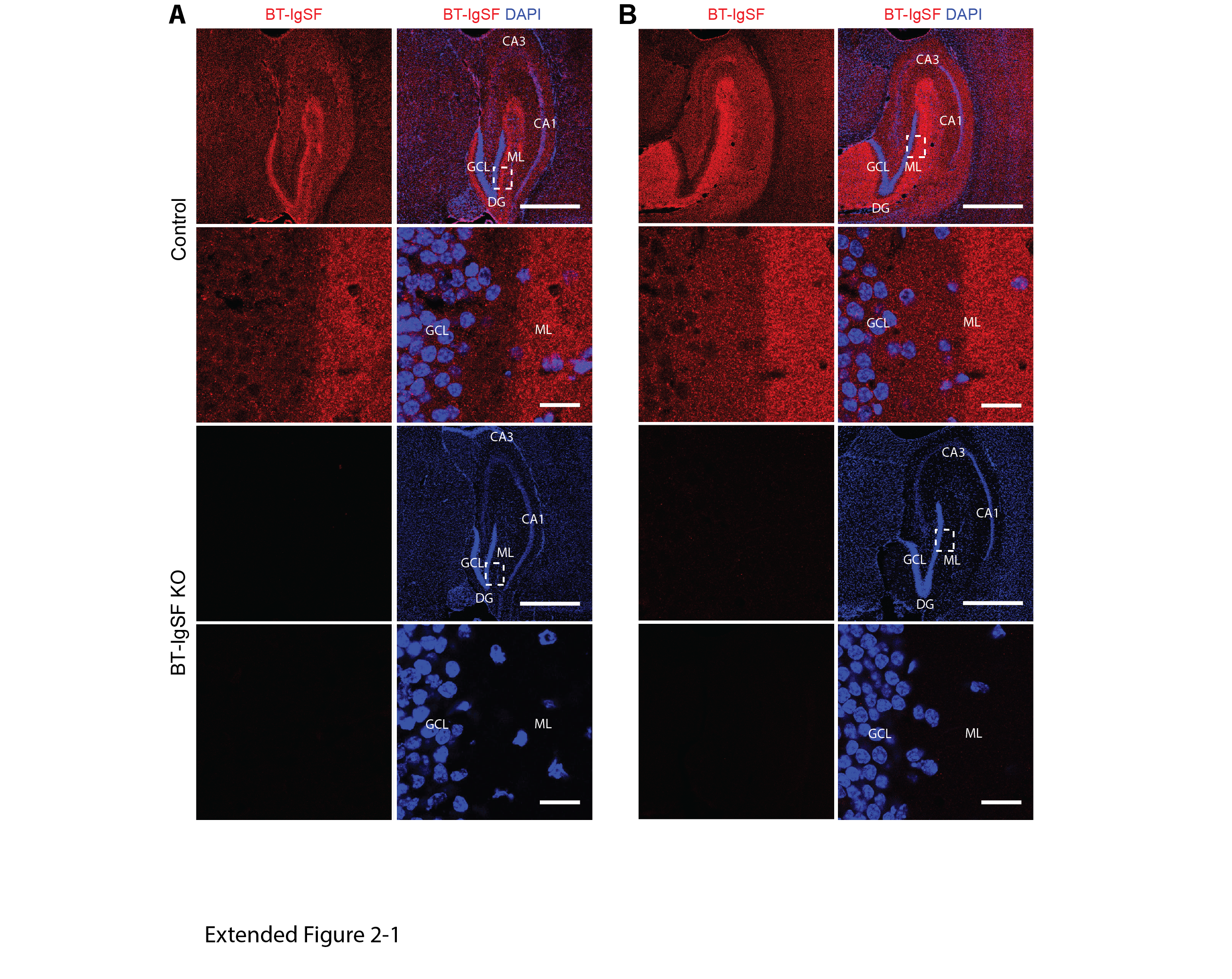

Supplement: Figure 2-1 — Localization of BT-IgSF in the hippocampus at P14 and P80 and demonstration of the specificity of rabbit anti-BT-IgSF. Coronal cryostat sections of the hippocampus at P14 (A) or P80 (B) were stained with anti-BT-IgSF (Rb95; 1 µg/ml). The dashed boxes indicate the position of the enlarged region shown below row 1 and 3. Absence of staining in BT-IgSF knockout tissues demonstrates specificity of rabbit antibodies to BT-IgSF. Two independent animals of each age were analyzed. CA1, cornus ammonis 1; CA3, Cornus ammonis 3; DG, dentate gyrus; GCL, granule cell layer; ML, molecular layer. Scale bar first and third row, 1 mm; scale bar second and fourth row, 20 µm. Download Figure 2-1, TIF file. [file eneuro-11-ENEURO.0283-23.2024-s001.tif]

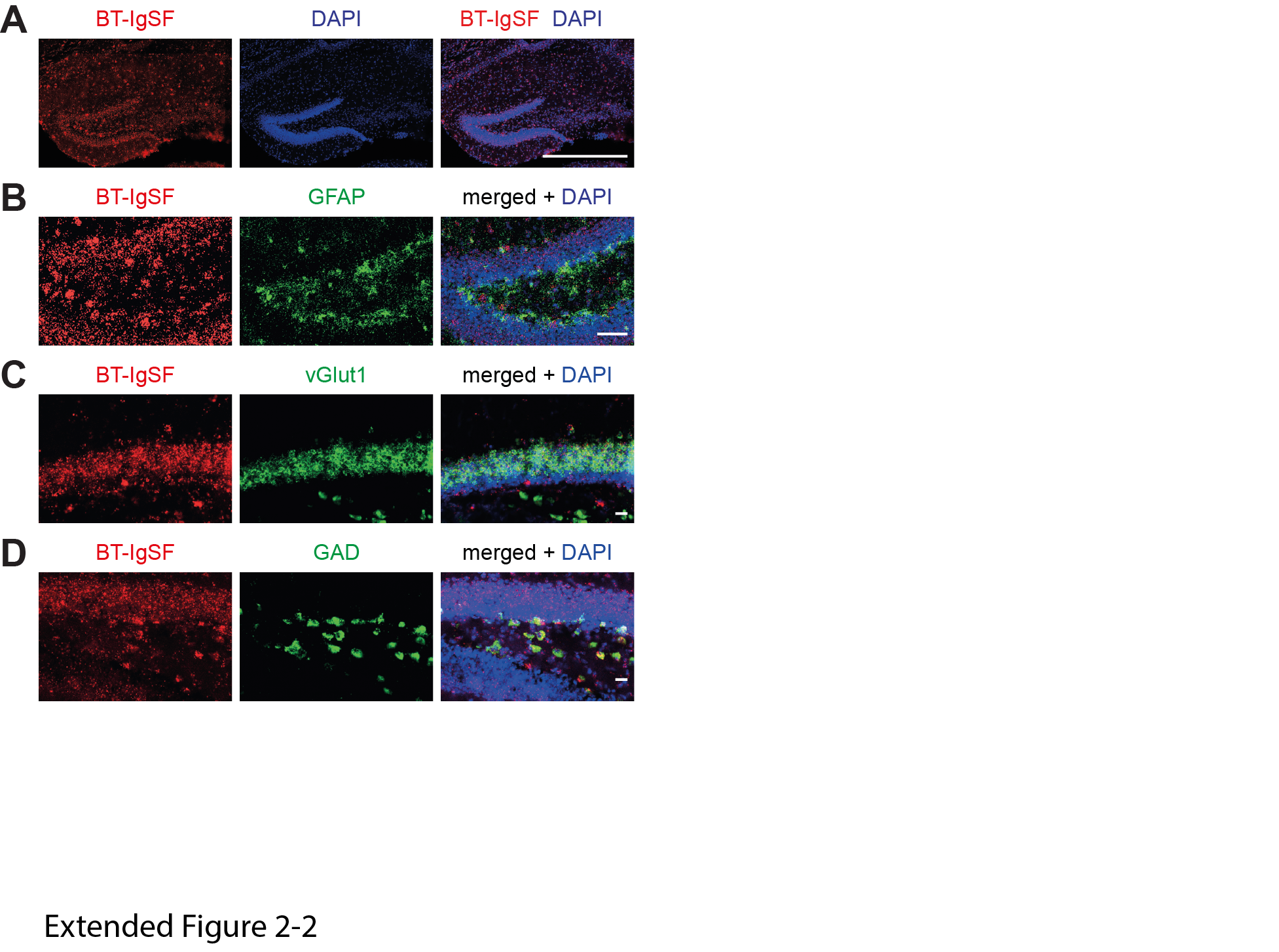

Supplement: Figure 2-2 — Bt-igsf encoding mRNA is expressed in excitatory and inhibitory neurons of the hippocampus. A) Overview of an RNA scope of Bt-igsf of a coronal section of a P20 hippocampus showing Bt-igsf mRNA encoding cells in all cell layers. Scale bar 500 µm. B) RNAscope of coronal sections of hippocampi at postnatal day 20 showing expression of Bt-igsf in GFAP-positive astrocytes (see also Figure 2F). C) RNAscope of coronal sections of hippocampi at postnatal day 20 showing expression of BT-IgSF in vGlut1-positive neurons. D) RNAscope of coronal sections of hippocampi at postnatal day 20 showing expression of Bt-igsf in Gad65-positive neurons. Three independent sections were analyzed. Download Figure 2-2, TIF file. [file eneuro-11-ENEURO.0283-23.2024-s002.tif]

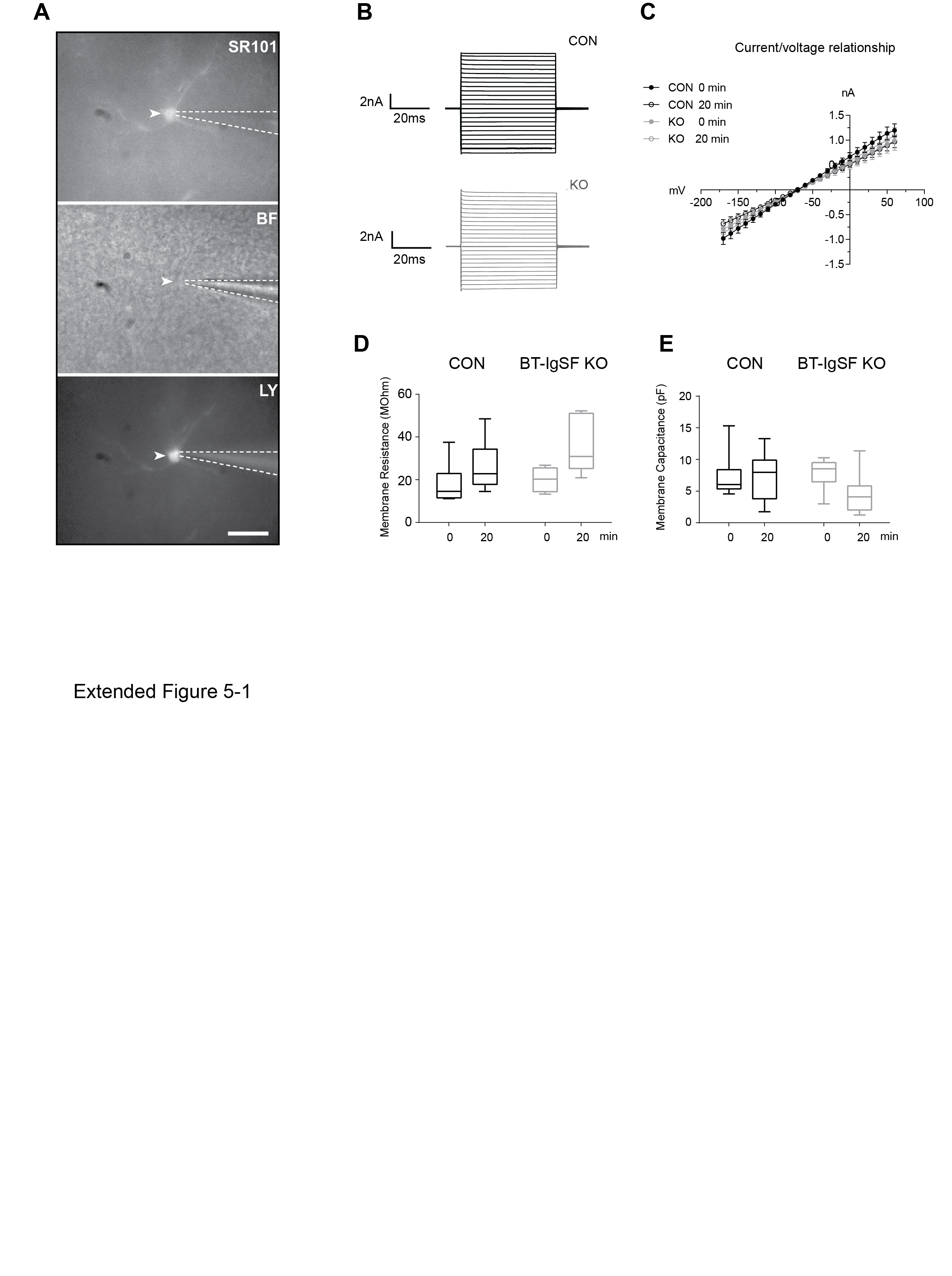

Supplement: Figure 5-1 — Analysis of dye spreading in astrocytes A) Bright field and fluorescence images of a patch-clamped astrocyte in the hippocampus. Top Sulforhodamine (SR101) staining of the patched astrocyte. The patch-clamp pipettes outline is marked as a dotted line. Middle Bright field (BF) image of the field of interest. The arrow marks the position of the patched astrocyte. Bottom To ensure success of the dialysis, Lucifer yellow (LY) was added to the pipette-solution. Bar, 20µm. B) Typical current profiles of an astrocyte in the wildtype (top) and knockout group (bottom) clamped at -70 mV in response to 10 de- and hyperpolarizing voltage-steps in hippocampus. Only cells which displayed a series resistance of ≤ 125% of the initial value after 20 min of dialysis were included in the analysis. C) The graph shows the averaged current to voltage relationship of both genotypes at the start and the end of the 20 min dialysis period in hippocampus (black: wildtype, grey: BT-IgSF knockout). D and E) The boxplot graphs compare the membrane capacitance and membrane resistance of hippocampal astrocytes at the start and the end of the dialysis. No significant differences were observed in either comparison (One-way ANOVA, p > 0.05). Data in C to E were compiled from dye spreading measurements. Similar results on the current profiles, membrane capacitance and membrane resistance were also obtained from cortical astrocytes. Download Figure 5-1, TIF file. [file eneuro-11-ENEURO.0283-23.2024-s003.tif]

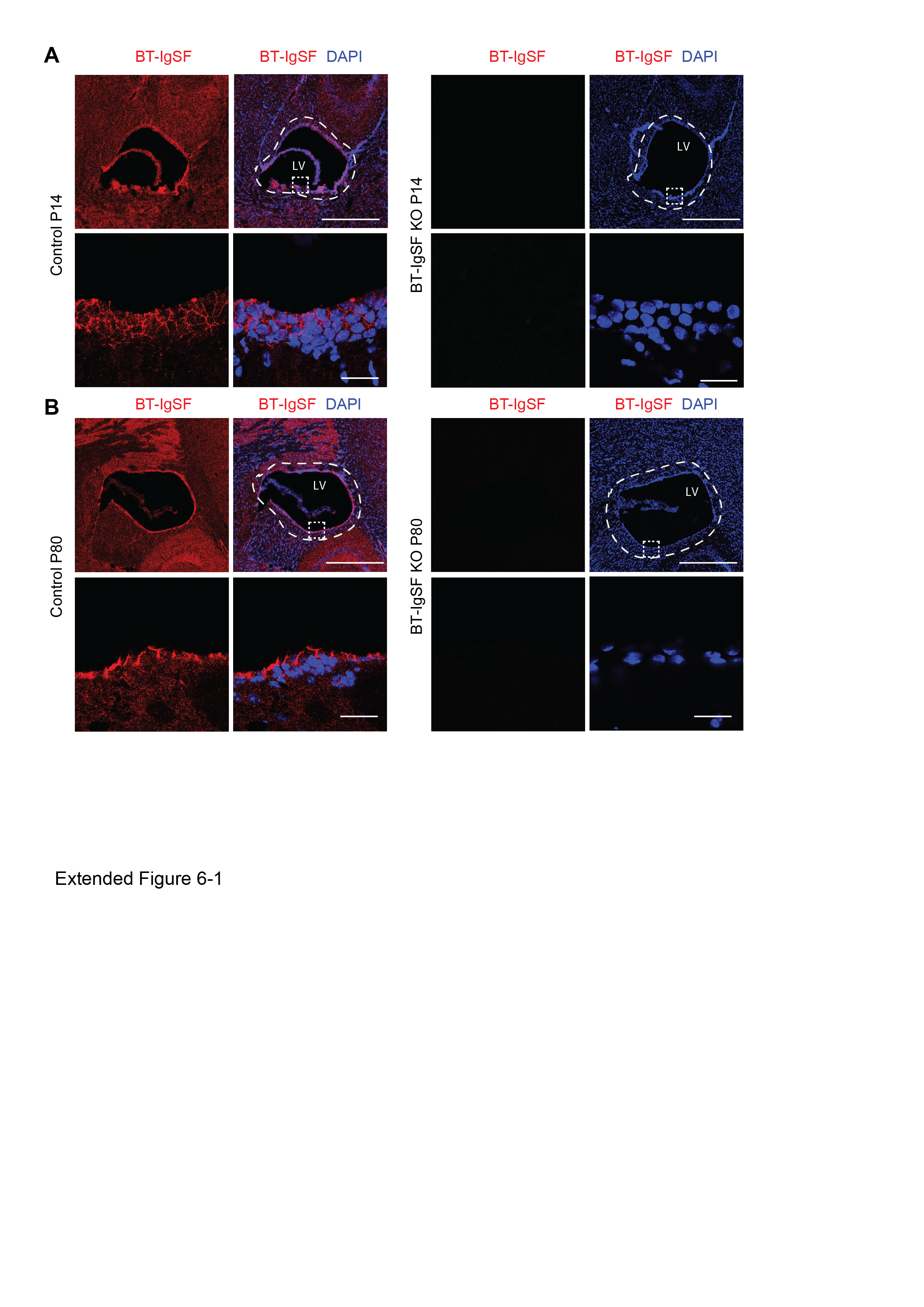

Supplement: Figure 6-1 — Localization of BT-IgSF at the lateral ventricle at P14 and P80. A and B) Coronal sections of the brain at P14 and P80 were stained with rabbit anti-BT-IgSF. BT-IgSF is strongly localized in cells lining the lateral ventricle. The dashed boxes indicate the position shown in row two or four. Absence of staining in BT-IgSF knockout tissues demonstrates specificity of antibodies to BT-IgSF. LV, lateral ventricle. Scale bar, first and third row, 1 mm; scale bar second and fourth row, 20 µm. Download Figure 6-1, TIF file. [file eneuro-11-ENEURO.0283-23.2024-s004.tif]
